# Hypoxia inducible factors regulate pneumovirus replication by enhancing innate immune sensing

**DOI:** 10.1073/pnas.2506647123

**Published:** 2026-04-07

**Authors:** Jiyeon Ha, Parul Sharma, Sammi Ta, Senko Tsukuda, James M. Harris, Rebekah Penrice-Randal, Eleanor Bentley, Adam Kirby, Daniele F. Mega, David A. Matthews, Peter Balfe, Jan Rehwinkel, Anja Kipar, James P. Stewart, Jane A. McKeating, Peter A. C. Wing

**Affiliations:** ^a^CAMS Oxford Institute, Chinese Academy of Medical Sciences & Peking Union Medical College, Nuffield Department of Medicine, University of Oxford, Oxford OX3 7BN, United Kingdom; ^b^Department of Infection Biology and Microbiomes, Institute of Infection, Veterinary and Ecological Sciences, University of Liverpool, Liverpool L3 5RF, United Kingdom; ^c^Nuffield Department of Medicine, University of Oxford, Oxford OX3 7FZ, United Kingdom; ^d^School of Cellular and Molecular Medicine, Faculty of Life Sciences, University of Bristol, Bristol BS8 1TD, United Kingdom; ^e^Medical Research Council Translational Immune Discovery Unit, Medical Research Council Weatherall Institute of Molecular Medicine, Radcliffe Department of Medicine, University of Oxford, Oxford OX3 9DS, United Kingdom; ^f^Laboratory for Animal Model Pathology, Institute of Veterinary Pathology, Vetsuisse Faculty, University of Zurich, Zurich 8057, Switzerland

**Keywords:** pneumovirus, HIF, innate immunity, hRSV

## Abstract

The immune mechanisms responsible for protection and pathogenesis in pneumoviral infection are not well defined. We demonstrated that pharmacological activation of the hypoxic inducible factor (HIF) signaling axis using daprodustat limited viral replication through enhanced immune signaling. Transcriptomic analysis revealed HIF augmented activation of innate immune response genes, including interferon-stimulated gene 15 (*Isg15*), in the lung and spleen of mice infected with pneumonia virus of mice (PVM). In human respiratory syncytial virus (hRSV)-infected airway epithelial cells, daprodustat inhibited viral replication and enhanced ISG15 expression in a HIF-dependent manner. Importantly, inhibition of type I interferon signaling or the RIG-I sensing pathway abrogated the antiviral activity of HIF. Moreover, daprodustat increased interferon signaling in response to viral RNA, suggesting that HIF inhibits pneumovirus replication through enhancing viral RNA sensing. Mechanistically, daprodustat reduced N^6^-methyladenosine modification of viral RNA through upregulation of RNA demethylases, promoting detection by innate immune sensors. This study highlights the intricate interplay between hypoxia and antiviral immunity and offers valuable insights into pneumovirus–host interactions and potential therapeutic interventions.

## Significance

This study explores the role of the hypoxic inducible factor (HIF) signaling pathway in limiting pneumoviral infections, such as respiratory syncytial virus (hRSV) and pneumonia virus of mice (PVM). Using daprodustat, a clinically approved activator of HIF signaling, we demonstrated that stimulation of this pathway enhanced innate immune signaling and limited viral replication. Transcriptomic analysis showed that HIF promoted innate immune response genes in mice infected with PVM and human airway epithelial cells infected with hRSV. Importantly, inhibition of type I interferon signaling or the RIG-I sensing pathway abrogated the antiviral activity of HIF. We uncovered a role for HIF to regulate the levels of N^6^-methyladenosine modification of viral RNA transcripts, resulting in the increased activation of nucleic acid sensing.

Pneumoviruses such as human respiratory syncytial virus (hRSV) cause lower respiratory tract illness in children (1), immunocompromised adults and the elderly (2). hRSV-induced bronchiolitis and pneumonia in infancy can lead to long-term respiratory sequelae, such as asthma (3). Most infections are mild; however, some infants develop a life-threatening inflammatory disease of the lower airways, the underlying causes of which are unknown. In low-middle-income countries, hRSV is the second most common cause of infant death after malaria (4, 5). Treatment options are limited, although the recent approval of hRSV-fusion protein vaccines for the elderly and pregnant women (4) provides opportunities to limit disease burden. Like other viruses, hRSV evolves resistance to immune and antiviral therapies (6–8), highlighting the need for a range of therapeutic approaches.

The airway epithelium is the first point of contact for hRSV, where infection is sensed by pattern recognition receptors (PRR), including Toll-like receptors (TLRs) and retinoic acid–inducible gene 1 (RIG-I)-like receptors (RLRs), which act in concert to trigger type-I interferon (IFN) expression to limit viral replication and spread (9). However, hRSV has developed effective countermeasures to circumvent host innate sensing and encodes several proteins that perturb multiple aspects of immune signaling. The effectiveness of these strategies is highlighted by the low to undetectable levels of type I IFN in nasal washes collected from infants with severe hRSV infection, in stark contrast to influenza A or parainfluenza virus infection (10–12). Mononuclear cells obtained from infants with severe bronchiolitis express low levels of IFN-α (12) and ex vivo infection of human macrophages or peripheral blood mononuclear cells with hRSV leads to modest type I IFN expression (13). Somewhat paradoxically, hRSV-infected lung epithelial cells from infants have been reported to express high levels of IFN-stimulated genes such as ISG15, a ubiquitin-like protein with immunomodulatory properties, mediated through a process known as ISGylation (14). Notably, an association between high levels of hRSV RNA and ISG15 expression in nasal aspirates was reported in infected infants, highlighting the potential use of ISG15 as a biomarker of hRSV-induced inflammation (15).

Among the various hRSV proteins involved in immune evasion, the best characterized are the nonstructural (NS) 1 and 2 proteins (16). The NS1-2 open reading frames (ORFs) are positioned proximal to the 3’ leader region within the viral genome, making them the earliest expressed and most abundant viral transcripts within an infected cell (17, 18). Indeed, these two NS proteins distinguish hRSV and PVM from other members of the *Mononegavirales*. NS1 and NS2 antagonism of type I and III IFN production has been extensively studied and is supported by the attenuation of viral replication in strains that lack either isoform in both in vivo and in vitro replication models (19–21). Notably these proteins disrupt the interaction between RIG-I and mitochondrial antiviral signaling protein (MAVS), either by direct binding to MAVS (22) or by targeting TRIM25 (23). The hRSV nucleoprotein (N), an integral component of the viral nucleocapsid, forms decameric rings around the single-stranded (ss) RNA genome (24, 25) and limits the recognition by cellular PRRs. N also sequesters innate signaling proteins into multimeric biomolecular condensates termed inclusion bodies (IBs) that are formed by the N and phosphoprotein (P) that drives liquid phase separation to generate IBs (26–28). Notably, these cytoplasmic structures are sites of viral replication, where viral proteins and replicase complexes are located (27). Recent work has shown that IB-associated granules concentrate nascent viral RNA complexes and components of the eukaryotic translation machinery to drive translation initiation (29). In early replication, MAVS and MDA5 are sequestered to small IBs through interactions with N, thereby inhibiting their antiviral function(s) (30), demonstrating how hRSV perturbs the spatial distribution of host antiviral defenses. The multifaceted approach by which hRSV evades and manipulates the host immune responses highlights the complexity of virus–host interactions and underscores the challenges in developing effective interventions against this pathogen.

While hRSV employs sophisticated mechanisms to evade host immune responses, our understanding of the cellular factors that influence viral replication and inflammation is more limited. In this context, hypoxia-inducible factors (HIFs) have emerged as important regulators of both viral infection and inflammatory responses in the lower respiratory tract (31, 32). HIFs are conserved transcription factors that define the response of the transcriptome and metabolome to changing oxygen levels (33). The three isoforms (HIF-1α, HIF-2α and the lesser studied HIF-3α) are regulated by prolyl-hydroxylase domain (PHD) enzymes whose activity depends on oxygen, iron, and 2-oxoglutarate. When oxygen is abundant, hydroxylation of two specific prolyl residues in the HIF-α subunits promote interaction with the von Hippel–Lindau ubiquitin (VHL) E3 ligase leading to ubiquitylation and proteasomal degradation (34–36). When oxygen is limited, this activity is suppressed and HIF-α subunits dimerize with HIF-1β, forming an active transcriptome complex. Through interactions with cellular importins, these complexes translocate to the nucleus and drive transcription through interaction with a consensus RCGTG(C) motif, also known as hypoxic responsive element (HRE), in the promoter and enhancer regions of responsive genes (37). Additionally, nonhypoxic stimuli, including metabolic and inflammatory signals, can regulate HIF expression (38, 39). HIF-regulated genes vary between cell types and silencing HIF-1α/-2α reveals their unique transcriptional signatures, highlighting tissue- and isoform- specific responses to these diverse physiological signals (40). Clinically, several PHD inhibitors (PHDi) are licensed for treatment of anemia (41). Our recent studies show a dual role for HIFs in restricting hRSV and SARS-CoV-2 infection and inflammatory responses (42–44), providing opportunities to exploit current licensed HIF-mimetic drugs for the treatment of respiratory infections.

Animal models of human RSV (hRSV) are limited by the semipermissive nature of many rodent and nonhuman primates (45). Chimpanzees are the exception and replicate comparable levels of hRSV to human infants (46, 47), which have been used to evaluate the virulence and protective efficacy of live attenuated vaccine candidates (48). BALB/c mice show intermediate susceptibility to experimental hRSV infection (49), and as such high infectious doses are required to induce clinical signs of disease, such as weight loss, ruffled fur, and hunching (50, 51). One model to study pneumovirus infection is the natural mouse pathogen, pneumonia virus of mice (PVM) (52), which is associated with severe morbidity, weight loss, labored breathing and mortality (53, 54). The virus replicates predominantly in alveolar and bronchial epithelial cells, inducing increased eosinophil counts in bronchial alveolar lavage samples, followed by a dominant neutrophil response (55, 56). Natural in vivo models of viral disease such as PVM provide valuable insights into pneumovirus pathogenesis and serve as a platform to evaluate potential therapeutic interventions.

In this study, we explored the impact of HIF signaling on pneumoviral disease by treating PVM-infected mice with the licensed PHDi, daprodustat to activate HIF signaling. Daprodustat inhibited PVM replication and immune infiltration and promoted pulmonary innate-immune signaling. Using in vitro model systems of hRSV infection, we confirmed these findings and demonstrated that the antiviral effect of HIF on hRSV replication is dependent on RIG-I signaling. Collectively, these findings highlight how targeting HIF-regulated pathways may offer an alternative approach for treating hRSV infection and the associated immune response.

## Results

### PVM Causes a Widely Disseminated Pulmonary Infection and Innate Immune Response.

**Fig. 1.**
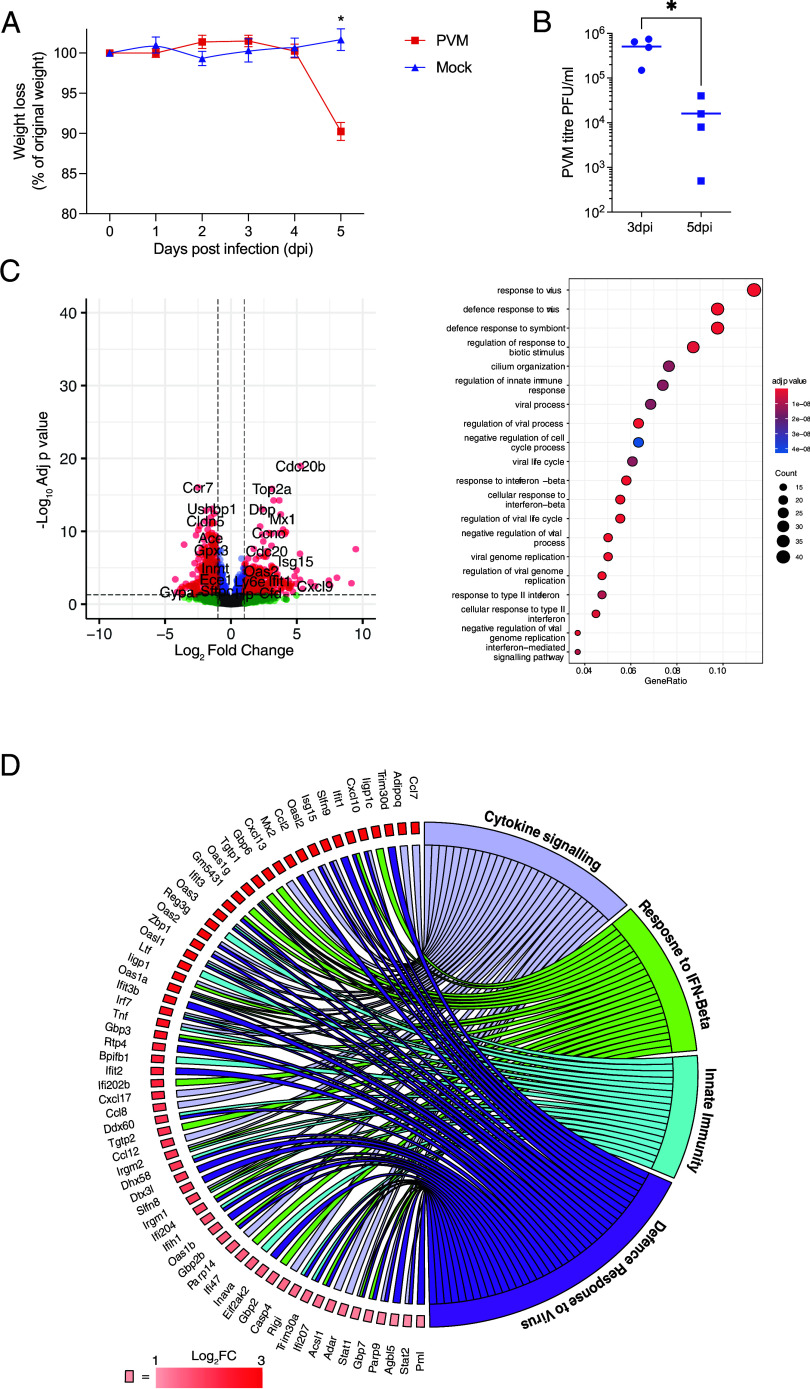
Characterizing PVM infection of the lung in mice. (*A*) Mice (n = 4) were infected intranasally with 1 × 10^4^ PFU of PVM-J3666 or PBS and body weight assessed daily up to 5 dpi. Data are mean ± SD of n = 4 animals. (*B*) Viral titers from lung tissue of infected mice measured at 3 dpi. (*C*) RNA sequencing analysis of uninfected or PVM-infected lungs at 3 dpi. The volcano plot depicts differentially expressed genes (DEGs) with thresholds defined as a log_2_ fold change of >1 or <−1 with an adjusted *P* value of <0.05 (dashed lines). The symbol color is derived from −log_10_ of the adjusted *P* value, where red indicates a log_2_ fold change of >1 or <−1 with an adjusted *P* value of <0.05. Up-regulated genes were subjected to gene-ontology analysis, with the top 20 enriched pathways depicted. Size of symbol indicates the number of genes from each pathway enriched in PVM infection. The gene ratio is the relative number of genes in a GO term that are enriched out of the total number of DEGs in the dataset. (*D*) Modified circos plot showing the relationship between the up-regulated genes from (*C*) and enriched GO pathways. Boxes adjacent to the gene labels are colored according to the log_2_ fold change of the individual gene between the infected and uninfected mice.

To study PVM infection in the lower respiratory tract, female BALB/c mice were inoculated intranasally with PVM strain J3666 at a dose of 1 × 10^4^ PFU per animal. We selected BALB/c mice given previous reports that this strain showed increased susceptibility to PVM compared to C57BL/6 mice (52). The infected mice maintained their body weight before showing a rapid loss at 5 d postinfection (dpi) (Fig. 1*A*). Infectious virus was detected in the lung tissue at 3 dpi (~5 × 10^5^ PFU/mL) and this declined to approximately 1.5 × 10^4^ PFU/mL at 5 dpi (Fig. 1*B*). Histological examination and staining for PVM-G protein revealed widespread antigen expression in the lung at 3 dpi, represented by multifocal patches of alveoli with type I and II pneumocytes and respiratory epithelial cells expressing the viral antigen along the luminal border in some bronchioles (*SI Appendix*, Fig. S1*A*). The associated histological changes were limited to focal areas of increased interstitial cellularity, with activated type II pneumocytes and mild perivascular mononuclear infiltration (*SI Appendix*, Fig. S1*A*). By day 5, viral antigen expression was apparent in the alveoli with large focal areas containing desquamated alveolar macrophages/type II pneumocytes and some leukocytes, consistent with acute pneumonia. There was evidence of leukocyte recruitment into the parenchyma, with leukocyte rolling and mild perivascular accumulation (*SI Appendix*, Fig. S1*B*). Minimal pathological changes were observed in either the parenchyma or alveoli of mock-infected mice (*SI Appendix*, Fig. S1*C*). To determine whether PVM had spread beyond the lungs, we examined the heart, thymus, spleen, liver, and kidney and found negligible evidence for any histological changes or viral antigen expression, in line with previous reports (57).

To elucidate the underlying molecular mechanisms driving the host antiviral response, we sequenced RNA from the lung of mock or PVM-infected animals at 3 dpi before the onset of infection-associated weight loss and distinct pathological changes. PVM induced a substantial change in the pulmonary transcriptome, with gene ontology (GO) analysis showing an enrichment of innate and immune-response pathways, indicative of a widely disseminated pulmonary infection, in agreement with previous studies (58) (Fig. 1*C*). We examined the relationship between the differentially expressed genes (DEGs) in response to PVM infection and the enriched immune pathways identified in Fig. 1*C*. This highlighted a key set of chemokine (*Cxcl9, Cxcl10,* and *Cxcl13*) and innate immune response genes (*Mx2, Oas2, Tnf, Ifit1,* and *Isg15*) that were up-regulated in PVM infection (Fig. 1*D*), underscoring the robust induction of host immune pathways elicited by PVM infection in the lungs.

### Daprodustat Limits PVM Infection and Associated Pathological Changes.

**Fig. 2.**
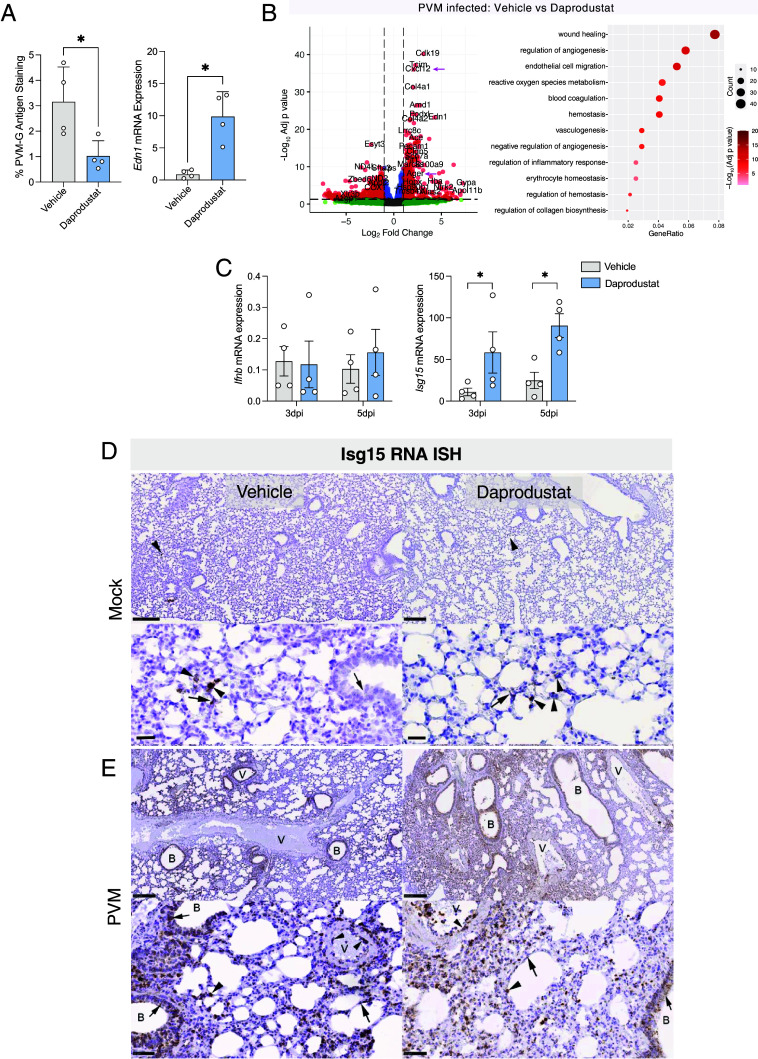
Impact of daprodustat treatment on PVM infection. (*A*) Quantification of PVM-G expression and RT-qPCR quantification of Endothelin-1 gene expression in lungs from PVM-infected mice treated with vehicle or daprodustat (30 mg/Kg) at 3 dpi. (*B*) Differential gene expression analysis of daprodustat-treated, PVM-infected animals compared to infected mice treated with vehicle. DEGs are defined as a log_2_ fold change of >1 or <−1, with an adjusted *P* value of <0.05. Up-regulated genes were used for gene-ontology analysis, with the top 12 enriched pathways depicted. (*C*) Quantification of *Ifnb* and *Isg15* mRNA by RT-qPCR from lung tissue harvested at 3 and 5 dpi from vehicle and daprodustat-treated groups. Data are expressed relative to the *Actb* housekeeper gene. (*D*) Detection of *Isg15* mRNA expressing cells in the lungs of vehicle (*Left* column) and daprodustat-treated (30 mg/Kg, *Right* column) mock-infected mice. The rare small patches of positivity in type I (arrows) and type II (arrowheads) pneumocytes in both treatment groups are annotated. A low signal was detected in some respiratory epithelial cells in the bronchiole (small arrow). (*E*) *Isg15* mRNA expression in PVM-infected mice treated with vehicle or daprodustat at 5 dpi. Widespread *Isg15* mRNA expression is seen after PVM infection in the bronchioles and parenchyma. Small arrows highlight expression in bronchiolar epithelial cells with large arrows and arrowheads denoting strong expression in type I and II pneumocytes, respectively. B: bronchiole; V: vessel. (Scale bar, 50 µm.)

As HIF signaling was previously shown to modulate the immune response (59) we investigated the therapeutic effect(s) of daprodustat, a HIF-PHDi, on PVM infection. PVM-infected mice were treated 24 h postinfection with 30 mg/Kg of daprodustat or vehicle by oral gavage for 3 or 5 dpi. Given the decline in infectious viral titer noted between 3 and 5 dpi (Fig. 1*B*), we examined the impact of daprodustat on viral replication at 3 dpi. The cell types expressing viral antigen were comparable in both groups; however, their frequency was lower following daprodustat treatment, with fewer bronchioles staining for PVM-G and smaller patches of infected alveolar epithelial cells (*SI Appendix*, Fig. S2*A*). This observation was confirmed by a quantitative analysis of PVM-G expression in the lung (Fig. 2*A*). To confirm drug efficacy, we quantified the HIF-target gene endothelin 1 (*Edn1*) (60) in the lung and noted a significantly higher expression in daprodustat-treated animals (Fig. 2*A*). To further investigate the effects of daprodustat, we conducted transcriptomic analysis of lung tissue from mice at 3 dpi. This revealed an enrichment of wound healing, angiogenic, and hemostatic pathways (Fig. 2*B*), in agreement with our previous work using the HIF-PHDi Roxadustat in a rodent model of SARS-CoV-2 infection (43).

Treatment of infected mice with daprodustat resulted in a significant increase in inflammatory gene expression such as *Cxcl12* and *Ager* (Fig. 2*B*), prompting further investigation of the composition and extent of immune cell infiltration in the lung tissue. We selected samples at 5 dpi for detailed histological analysis, considering the more substantial pathological changes observed at this timepoint (*SI Appendix*, Fig. S1*B*). Examination of these tissues revealed focal areas of consolidation in the lung, with desquamation of alveolar epithelia/alveolar macrophages and leukocyte infiltration in the alveolar lumina (*SI Appendix*, Fig. S2*B*). Vehicle-treated animals exhibited emigration and perivascular accumulation of mononuclear leukocytes around blood vessels, in contrast to the daprodustat-treated mice. Given the increased expression of chemokine and cytokine genes in response to PVM infection from our earlier transcriptomic analysis (Fig. 1 *C* and *D*), we assessed the extent of leukocyte recruitment and composition of perivascular infiltrates in both vehicle and drug treated groups. Lung sections were immunostained to visualize T cells (CD3+), monocytes/macrophages (Iba1+), and neutrophils (Ly6G+). Vehicle treated mice showed extensive monocyte/macrophage recruitment into the lung parenchyma (*SI Appendix*, Fig. S2*B*). T cells were less abundant, representing individual cells in the lumen of capillaries and in inflammatory infiltrates. In contrast, in the PVM-infected daprodustat-treated mice, there was limited evidence of monocyte/macrophage recruitment and infiltration, accompanied by few T cells (*SI Appendix*, Fig. S2*B*). Neutrophils were more prevalent and identified within capillaries and in regions of alveolar damage and infiltration, with comparable quantities in both groups (*SI Appendix*, Fig. S2*B*). These findings highlight the differential immune cell recruitment and infiltration patterns in PVM-infected mice treated with daprodustat compared to vehicle-treated controls, suggesting an immunomodulatory role for HIFs in the regulation of inflammatory response during viral infection.

To assess the impact of HIF on the host response to PVM, we performed targeted gene expression analysis of *Ifnb* and *Isg15* transcripts by RT-qPCR from vehicle and daprodustat-treated groups. Daprodustat augmented *Isg15* expression but had a minimal effect on *Ifnb* (Fig. 2*C*). We also examined the spatial distribution of *Isg15* transcripts in the tissue samples using RNA in situ hybridization (ISH), noting limited expression in the lungs of uninfected mice, irrespective of daprodustat treatment (Fig. 2*D*). However, in the spleen, moderate numbers of *Isg15-*positive cells were detected in the red pulp of uninfected animals treated with vehicle, with a modest increase following daprodustat treatment (*SI Appendix*, Fig. S3*A*). Extensive *Isg15* mRNA expression was observed in the lungs of the PVM-infected mice at 5 dpi (Fig. 2*E*) with alveolar and bronchiolar epithelial cells, as well as infiltrating leukocytes and vascular endothelial cells, showing high levels of *Isg15* expression which was more extensive following daprodustat treatment (Fig. 2*E*). In PVM-infected mice the frequency of *Isg15* mRNA expressing cells in the splenic red pulp was substantially increased and this was more pronounced following daprodustat treatment (*SI Appendix*, Fig. S3*B*). To assess the cell types expressing *Isg15* in the lung, sections were costained with the monocyte/macrophage marker Iba1 or the neutrophil marker Ly6G, demonstrating *Isg15* expression in monocytes, infiltrating/alveolar macrophages, and neutrophils, respectively (*SI Appendix*, Fig. S3*C*). Together, these tissue-specific and infection-dependent effects of daprodustat on *Isg15* expression highlight a role for HIFs in regulating the innate immune response to PVM infection.

### Daprodustat Promotes Innate Immune Gene Expression in PVM Infection.

**Fig. 3.**
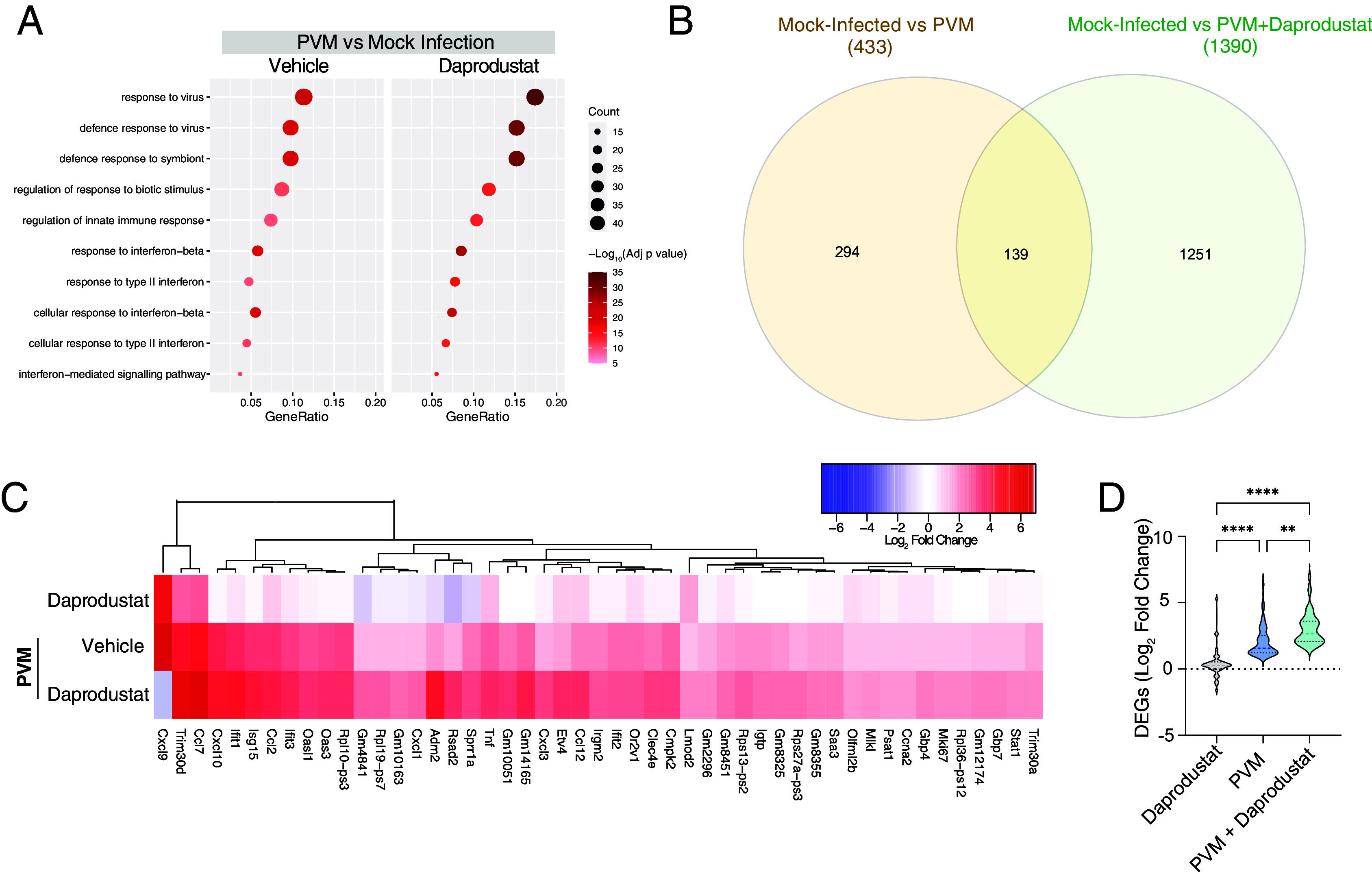
Daprodustat augments innate immune responses to PVM. (*A*) Differential gene expression analysis of daprodustat-treated PVM-infected animals compared to the vehicle control group. Comparison of enriched GO pathways in uninfected and PVM-infected animals treated with vehicle or daprodustat. Symbol size represents the number of genes from each pathway enriched by vehicle or daprodustat treatment. The symbol color is derived from −log_10_ of the adjusted *P* value. The gene ratio is the relative number of genes in a GO term that are enriched out of the total number of DEGs for each dataset. (*B*) Venn diagram showing the intersection between up-regulated DEGs in PVM and PVM-daprodustat-treated animals compared with uninfected animals. (*C*) Hierarchical clustering of the 59 common innate immune genes (derived from *B*) in daprodustat-treated uninfected mice and PVM-infected mice treated with vehicle or daprodustat. Colors are derived from the log_2_ fold change in expression relative to uninfected, untreated animals, where red indicates a log_2_ fold change of >1 or <−1 with an adjusted *P* value of <0.05. (*D*) The log_2_ fold changes from the entire dataset from *C* were compared by ANOVA; **P < 0.01, ***P < 0.001, and ****P < 0.0001.

Building on our histopathological analysis of the lung tissue, we examined the broader transcriptomic changes induced by daprodustat during PVM infection. Daprodustat treatment resulted in an enrichment of innate-response pathways in PVM-infected mice, with higher gene ratios noted for each GO pathway (Fig. 3*A*). We therefore interrogated our RNA sequencing data with a focus on a consolidated list of innate response genes based on the significantly enriched innate immune GO pathways observed in vehicle-treated PVM-infected mice. Hierarchical clustering showed a significant increase in the expression of RNA editing enzymes, *Adar* and *Apobec3*, type I IFN response genes such as *Ifit1, Ifit2, Oas2, Oas3,* and *Isg15,* and PPR genes, including *Ddx58* (encoding RIG-I) and *Tlr3*, in PVM-infected animals compared to mock-infected controls (*SI Appendix*, Fig. S4*A*). Notably, daprodustat treatment enhanced the expression of innate genes, including an array of IFN genes, that were not observed in PVM infection alone, such as *Ifng, Ifna1, Ifna4, Tlr9,* and *Trim38* (*SI Appendix*, Fig. S4*A*). The increased expression of toll-like receptors such as *Tlr9* and *Tlr3* in daprodustat-treated infected mice suggests that HIFs may prime the innate immune response to detect a broad range of viral nucleic acids across the endosomal pathway. Mapping the potential cell types in the lung using digital cell quantification (61) suggests that daprodustat modulates the abundance of monocytes, dendritic, and NK cells (*SI Appendix*, Fig. S4 *B* and *C*). Notably, we observed reduced macrophage signatures in line with our immunohistochemical analysis of infected lung tissue (*SI Appendix*, Fig. S2*B*). Collectively, these findings suggest a wider impact of HIF regulation of the innate immune system to detect viral pathogens.

Analysis of the lung transcriptomic data from PVM-infected animals treated with vehicle or daprodustat identified 139 genes with significantly increased expression relative to mock-infected animals (Fig. 3*B*). To focus our analysis on innate response genes, we narrowed our investigation to a list of 59 genes, previously identified as innate immune response genes. Daprodustat significantly enhanced expression of this gene set in PVM infection and, importantly, we noted negligible modulation of these genes in uninfected mice treated with daprodustat, suggesting that PVM infection was a prerequisite to observe these changes in innate gene expression (Fig. 3 *C* and *D*).

### Daprodustat Inhibits hRSV Infection.

**Fig. 4.**
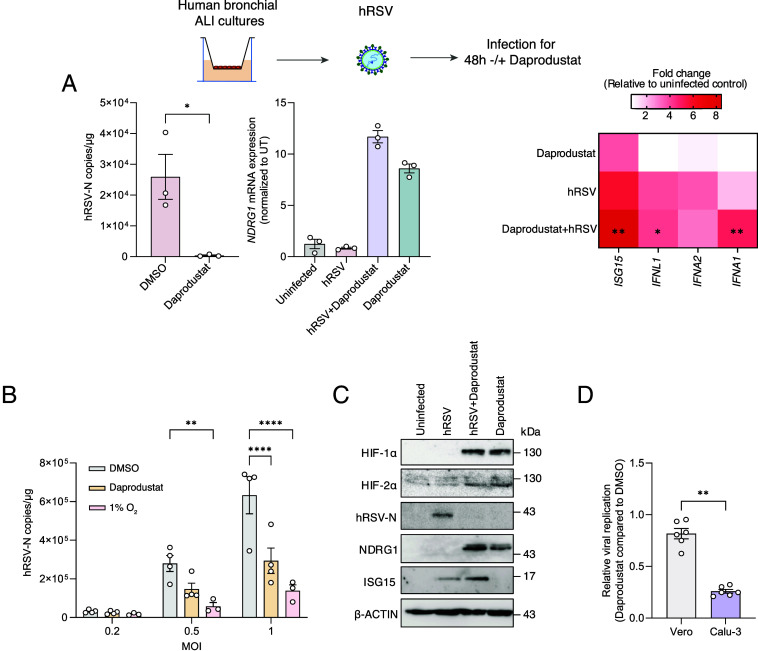
Daprodustat and low oxygen inhibit hRSV infection and increase ISG15 expression. (*A*) ALI-differentiated PBECs from 3 donors were infected with hRSV at an MOI of 1 and treated with daprodustat (50 µM) for 48 h. hRSV-N and expression of *NDRG1, ISG15, IFNL1, IFNA1,* and *IFNA2* were assessed by RT-qPCR. (*B*) Calu-3 were infected with hRSV at a range of MOIs and treated with either 50 µM of daprodustat or 1% O_2_ for 48 h. hRSV-N was quantified by RT-qPCR with statistical significance assessed by ANOVA. (*C*) Protein expression of HIF-1α and 2α, hRSV-N, NDRG1, and ISG15 in Calu-3 cells treated for 48 h with daprodustat with or without hRSV infection. (*D*) Vero or Calu-3 cells were infected with hRSV (MOI 1) and treated with 50 µM of daprodustat for 48 h. Viral transcripts were quantified by RT-qPCR and compared relative to their DMSO control to determine the relative antiviral effect of the drug treatment. Unless otherwise stated, plotted points indicate independent biological replicates and shown as mean ± SD. Statistical significance was determined by ANOVA; **P < 0.01, ***P < 0.001, and ****P < 0.0001.

We investigated whether the elevated expression of innate antiviral genes such as *Isg15, Ifnl1, Ifna-1,* and *-4* in the daprodustat-treated, PVM-infected mice extended to hRSV. Air–liquid interface cultures of human primary bronchial epithelial cells (ALI-PBEC) were used to assess the impact of daprodustat on innate response gene expression following hRSV infection. Daprodustat treatment resulted in a significant reduction in hRSV-N gene expression and a concomitant increase in the HIF-target gene N-myc downstream-regulated gene 1 (*NDRG1*) expression (Fig. 4*A*). In line with our PVM-infected lung RNA-seq (*SI Appendix*, Fig. S4), daprodustat increased innate antiviral gene expression (Fig. 4*A*). To extend this observation, we infected Calu-3 lung epithelial cells with increasing doses of hRSV followed by daprodustat treatment or cultured under low oxygen conditions (1% O_2_) previously reported to induce HIF expression (42). Both treatments significantly reduced hRSV-N gene and protein expression (Fig. 4*B*). Stabilization of both HIF-1 and HIF-2α isoforms and induction of NDRG1 confirmed daprodustat activity (Fig. 4*C*). We detected increased levels of ISG15 in hRSV-infected cells treated with daprodustat, confirming our observations with ALI-PBECs and the in vivo data obtained from PVM-infected mice (Fig. 4*C*). Comparing the impact of either daprodustat or hypoxia on PVM replication in baby hamster kidney (BHK) cells, which support PVM replication (62), showed that both treatments significantly reduced PVM N and NS2 gene expression, as well as the infectious viral load (*SI Appendix*, Fig. S5 *A*–*C*). To assess whether hRSV infection induced IFN signaling, we incubated the extracellular media from infected HEp-2 cells, a well-described hRSV-permissive cell line (63), with HEK-293 cells expressing a luciferase-based interferon-stimulated response element (ISRE) reporter construct. hRSV activated the ISRE reporter, with daprodustat significantly enhancing this effect, indicative of increased type I IFN production (*SI Appendix*, Fig. S5*D*). To assess whether daprodustat inhibition of hRSV replication is dependent on innate immune signaling, we evaluated the antiviral capacity of daprodustat in Vero cells, which lack the ability to produce endogenous IFN (64), but exhibit comparable responses to daprodustat as assessed by *NDRG1* expression (*SI Appendix*, Fig. S5*E*). Vero cells were highly permissive for hRSV replication; however, the inhibitory activity of daprodustat was diminished compared to Calu-3 cells, suggesting that IFN signaling is a major component of the antiviral activity of daprodustat (*SI Appendix*, Fig. S5*E*).

**Fig. 5.**
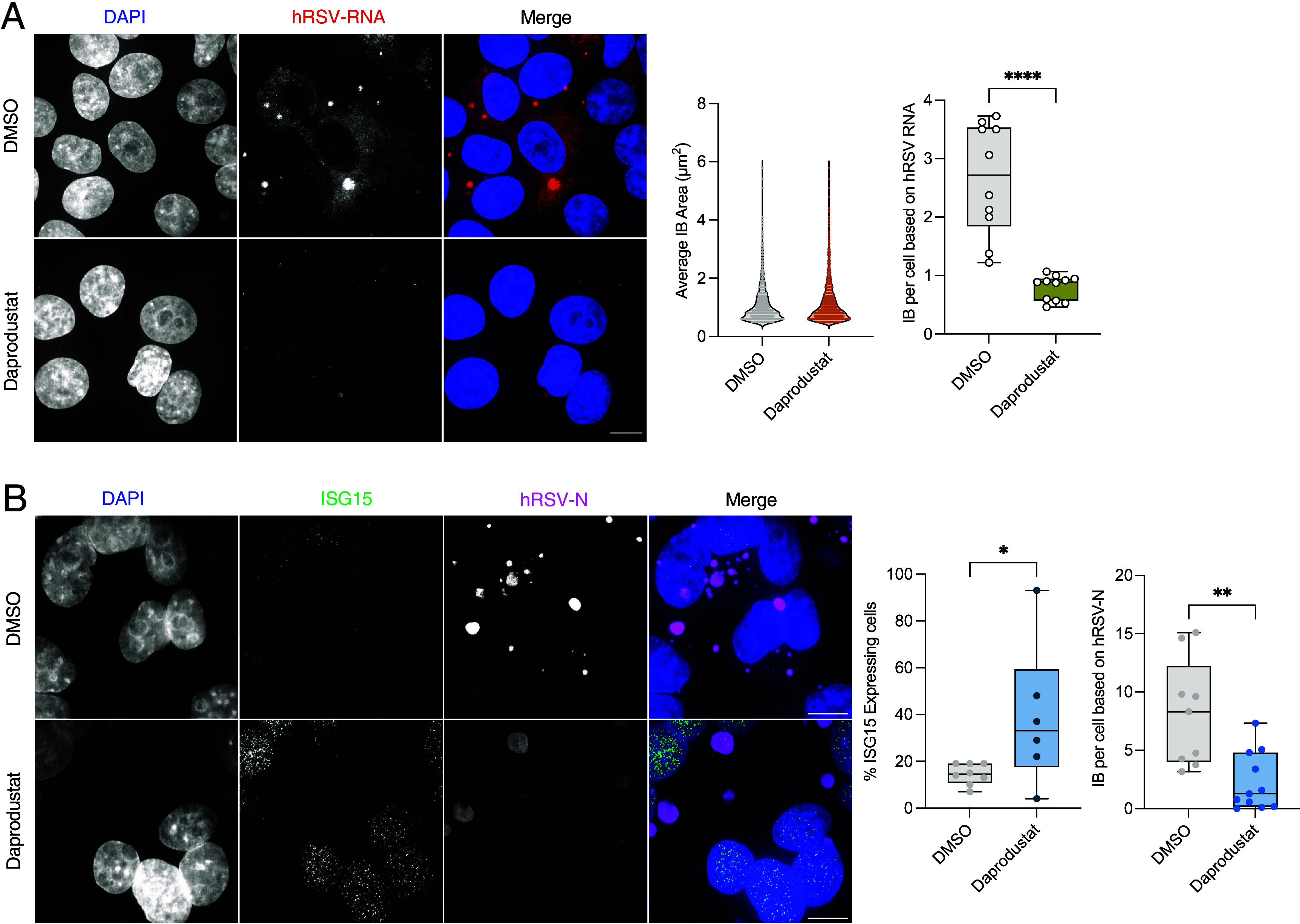
Daprodustat reduces the number of IBs and enhances nuclear ISG15 expression. (*A*) HEp-2 cells were infected with hRSV (MOI 1) and treated with daprodustat (50 µM) for 48 h. Viral RNA was visualized through fluorescent in situ hybridization using directly conjugated probes targeting hRSV-N and P transcripts. The area and number of viral IBs per cell were quantified. Individual channels are shown together with merged color images. (Scale bar, 10 µm.) (*B*) ISG15 expression was visualized in hRSV-infected HEp-2 cells treated with or without 50 µM of daprodustat by immunofluorescent staining. Cells were also stained for the hRSV-N protein to assess viral replication. (Scale bar, 10 µm.) The percentage of ISG15-positive cells per field of view was quantified, as was the average number of viral IB per cell. Statistical significance was determined by Student’s *t* test; *P < 0.05 and **P < 0.01.

An essential characteristic of hRSV infection at the subcellular level is the formation of IBs that regulate the spatiotemporal compartmentalization of viral RNA replication (29). smFISH imaging showed that hRSV-N and P transcripts principally localize within cytoplasmic IB-like structures, as previously reported (27) (Fig. 5*A*). Daprodustat treatment reduced the number of IBs, although treatment had no discernible impact on their average size (Fig. 5*A*). We assessed the intracellular levels of ISG15 in hRSV-infected cells and noted significantly increased nuclear expression upon daprodustat treatment in hRSV-N expressing cells (Fig. 5*B*). These data support our earlier in vivo findings that daprodustat enhances ISG15 and innate immune induction in hRSV infection, demonstrating the specificity of this finding in virus-infected cells.

### Daprodustat Inhibition of hRSV Is HIF Dependent.

**Fig. 6.**
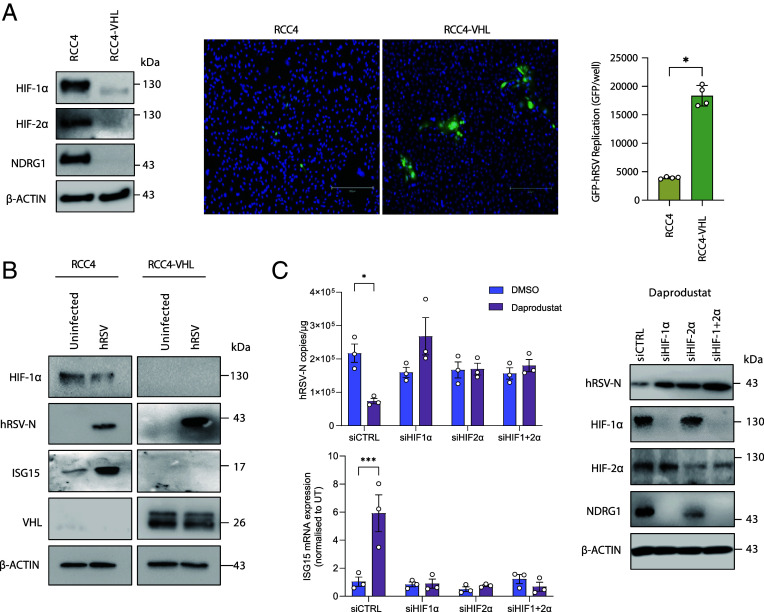
HIF-dependent restriction of hRSV. (*A*) RCC4 or RCC4-VHL cells were infected with GFP-hRSV at an MOI of 1 for 48 h. Expression of HIF-1α, HIF-2α and NDRG1 was assessed by immunoblot and GFP expression quantified by Clariostar. (*B*) Expression of ISG15, hRSV-N, HIF-1α, and VHL was assessed by immunoblot. (*C*) Calu-3 cells were transfected with siRNA targeting HIF-1α or HIF-2α either individually or in combination followed by infection with hRSV and treatment with 50 µM of daprodustat. Viral replication was assessed 48 h postinfection by RT-qPCR with quantification of hRSV-N transcripts and *ISG15* mRNA. Protein expression of HIF-1α, HIF-2α NDRG1, and hRSV-N was determined by immunoblot. Unless otherwise stated, points represent independent biological replicates and are plotted as mean ± SD. Statistical significance was determined by ANOVA, **P < 0.01, ***P < 0.001, and ****P < 0.0001.

To ascertain whether HIF directly impacts hRSV replication, we utilized the well-characterized renal carcinoma cell line RCC4 which lacks functional VHL and constitutively expresses HIF-1α and 2α under normoxic conditions (65). Overexpression of VHL in the RCC4 cells (RCC4-VHL) restored physiological degradation of HIF isoforms under normoxic conditions (Fig. 6*A*). GFP-hRSV replication was significantly reduced in RCC4 compared with RCC4-VHL cells, consistent with endogenous HIFs restricting viral replication (Fig. 6 *A* and *B*). ISG15 gene and protein expression were increased in RCC4 cells following hRSV infection compared to RCC4-VHL cells (*SI Appendix*, Fig. S6*A*). As an additional model, we used the renal cell line 786-0 cells, which lack both VHL and HIF-1α transcripts (65) allowing us to assess the role of HIF-2α on hRSV replication. We observed a similar reduction in hRSV N protein expression in the VHL expressing 786-0 cells compared to the wild-type cells (*SI Appendix*, Fig. S6*B*). In line with our RCC4 results, ISG15 expression was increased following hRSV infection, although to a lesser degree. To extend these findings, we performed siRNA knockdown of both HIF-1α and 2α and assessed the effect of daprodustat on viral replication in HIF depleted cells. Daprodustat had a reduced effect on viral replication when either HIF isoform was ablated (Fig. 6*C*), confirming a role for both HIF isoforms in regulating hRSV replication. Quantification of *NDRG1* expression, which is a HIF-1α and 2α coregulated gene (40), confirmed the functional ablation of HIF transcriptional activity (*SI Appendix*, Fig. S6*C*). In summary, these data indicate that both HIF isoforms play direct roles in restricting hRSV replication.

### Daprodustat Exerts Its Antiviral Effect on hRSV Through RLR and Type I IFN Signaling.

**Fig. 7.**
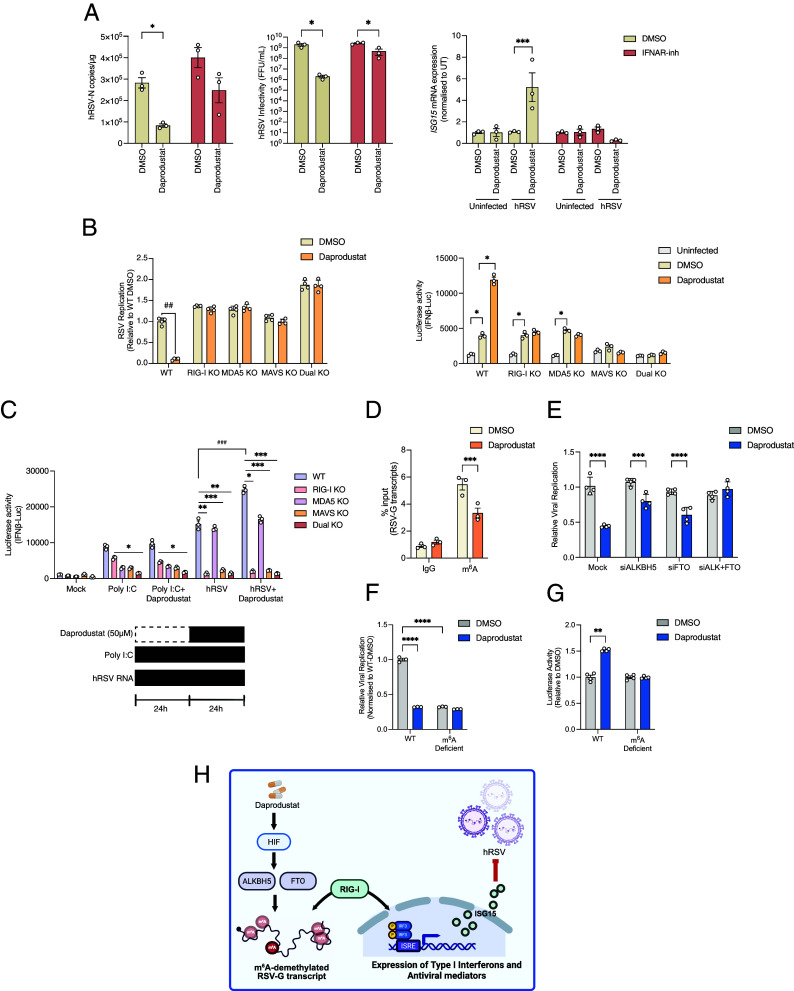
Daprodustat inhibition of hRSV replication is dependent on RIG-I signaling. (*A*) Calu-3 cells were infected with hRSV (MOI 1), treated with daprodustat (50 µM) with or without the IFN-alpha receptor inhibitor (10 µM) for 48 h. The infectious titer was assessed by FFU assay and hRSV-N and *ISG15* mRNA expression was determined by RT-qPCR. (*B*) HEK293-IFN-β luciferase reporter cells were infected with hRSV (MOI 1) and treated with 50 µM of daprodustat. IFN-β-promoter luciferase activity and hRSV-N expression by immunofluorescence were quantified at 96 h postinfection in WT and KO cells. (*C*) WT and KO cells were transfected with hRSV-particle-associated RNA or poly I:C and treated with or without daprodustat. Luciferase activity was quantified 48 h posttransfection. (*D*) Quantification of hRSV-G transcripts from m^6^A RNA immunoprecipitation from infected Calu-3 cells, expressed as % input of total RNA. RNA input was normalized, so an equal number of viral copies was used in the pull-down. (*E*) Calu-3 cells were transfected with siRNA against *ALKBH5* and *FTO*, either individually or in combination. Cells were infected with GFP-hRSV (MOI 1) and treated with daprodustat (50 µM). Viral replication was assessed after 48 h by quantification of GFP expression. (*F*) Calu-3 cells were infected with WT or m^6^A deficient hRSV (MOI 1) followed by treatment with daprodustat (50 µM) for 48 h. Viral replication was assessed after 48 h by quantification of GFP and data normalized to the DMSO control to assess the relative antiviral activity of the treatments. (*G*). HEK293-IFN-β luciferase reporter cells were transfected with hRSV RNA derived from WT or m^6^A-deficient hRSV stocks with or without daprodustat (50 µM) treatment. IFN-β promoter-driven luciferase activity was quantified 24 h posttransfection. (*H*) Schematic showing the proposed model of daprodustat regulation of m^6^A methylation increasing innate sensing of hRSV. Data are representative of 3 biological replicates with statistical significance determined by ANOVA; *P < 0.05, **P < 0.01, ***P < 0.001, and ****P < 0.0001.

Given the augmentation of *ISG15* and other ISGs in our transcriptomic analysis, we assessed the impact of daprodustat on IFN signaling in hRSV-infected Calu-3 cells over a 48 h period. We noted increased expression of ISG15, IRF3, and p-IRF3 within 24 h of hRSV infection in cells treated with daprodustat, but limited modulation of these proteins in polyinosinic-polycytidylic acid (Poly-I:C) treated cells (*SI Appendix*, Fig. S6*D*). To confirm the increased expression of p-IRF3 in Poly(I:C) stimulated cells 48 h following daprodustat treatment, we assessed IFN-β signaling activity using HEK293 cells stably expressing a firefly luciferase reporter under the control of the IFN-β promoter (HEK293-IFN-β-luciferase cells). We demonstrated that daprodustat significantly increased reporter activity in hRSV-infected cells (*SI Appendix*, Fig. S6*E*). To assess a direct role for IFN-signaling in the antiviral activity of daprodustat, we treated cells with a competitive antagonist of the IFN-alpha receptor (IFNAR-Inh) (66). IFNAR-Inh treatment inhibited STAT-1 phosphorylation in response to IFNα treatment confirming efficacy in Calu-3 cells (*SI Appendix*, Fig. S7*A*). The antiviral activity of daprodustat, assessed through hRSV N-gene expression and viral infectivity, was attenuated under IFNAR inhibition accompanied by reduced ISG15 expression, while showing no detectable effect on HIF-mediated transcriptional activity. (Fig. 7*A* and *SI Appendix*, Fig. S7*B*). To extend these findings, we showed that hRSV infection of IFN-alpha receptor knock out cells (IFNAR KO) was insensitive to the antiviral activity of daprodustat (*SI Appendix*, Fig. S7*C*). When assessing the IFN-β promoter activation of the supernatant of these cells, there was limited evidence for daprodustat-mediated increased activity in hRSV-infected IFNAR-KO cells (*SI Appendix*, Fig. S7*C*).

As daprodustat showed a reduced inhibition of hRSV infection in Vero cells (Fig. 4*D*), which do not produce endogenous IFN (64), we hypothesized that the antiviral phenotype is mediated via cell-intrinsic PRRs such as RIG-I or MDA5. To assess this, we infected HEK-293-IFN-β-luciferase WT, RIG-I, MDA5, double (RIG-I and MDA5), or MAVS KO cells (67) with a fluorescent GFP-hRSV, enabling the simultaneous measurement of viral replication and IFN induction. Increased levels of hRSV replication were observed in RIG-I and RIG-I/MDA5 dual KO cells compared to WT, demonstrating that these receptors restrict viral replication, and while daprodustat reduced viral replication in WT cells, this activity was lost in cells lacking RIG-I, MDA5, or MAVS (Fig. 7*B*). Quantification of IFN-β-driven luciferase confirmed increased activity in WT, RIG-I, and MDA5 KO cells on hRSV infection, which was not observed in MAVS or RIG-I/MDA5 dual KO cells (Fig. 7*B*). These data suggest that RIG-I and MDA5 play redundant roles in hRSV-induced activation of the IFN-β promoter in this setting. Notably, hRSV-infected WT cells treated with daprodustat exhibited a significant augmentation in reporter activity that was absent in the KO cells, suggesting that RIG-I and MDA5 signaling play a key role in HIF regulation of innate viral RNA-sensing pathways. To evaluate whether daprodustat increased the activity of other PRRs such as cGAS-STING or TLR3, we treated our IFN-β reporter line with the cGAS-STING agonist diABZI or cGAMP and showed a dose-dependent activation that was not influenced by daprodustat (*SI Appendix*, Fig. S7*D*). Next, we considered if this was a generic antiviral response and used Encephalomyocarditis virus (EMCV), a well-known activator of RLR signaling (68). Infection of our panel of KO cells with EMCV resulted in RIG-I and MDA5-dependent inductions of IFN-β promoter activity, which was not further enhanced by daprodustat treatment (*SI Appendix*, Fig. S7*E*).

To examine whether daprodustat increased detection of hRSV RNA through innate RNA sensing, we transfected our KO cells with RNA isolated from hRSV particles or with poly I:C, a positive control for RLR activation. While hRSV-RNA increased IFN-β reporter activity in the WT cells, this was diminished in the RIG-I, MAVS, and RIG-I/MDA5 dual KO cells but not in MDA5 KO cells, demonstrating an essential role for RIG-I to sense hRSV-RNA (Fig. 7*C*). Daprodustat treatment of hRSV-RNA transfected cells significantly increased IFN-β reporter activity in both the WT and MDA5 KO cells, but not in cell lines with ablated RIG-I or MAVS expression (Fig. 7*C*). Importantly, in poly I:C treated cells, daprodustat had a minimal effect on the IFN-β reporter activity. Collectively, these findings support a role for daprodustat to enhance the detection of hRSV RNA in a RIG-I-dependent manner.

Given the recent findings that loss of N^6^-methyladenosine (m^6^A) in hRSV-G transcripts resulted in increased detection of viral RNA by nucleic acid sensing (69) as well as HIF-regulated expression of the demethylase ALKBH5 reduces global m^6^A methylation (70), we hypothesized that HIF-driven depletion of RNA methylation would promote innate immune recognition of hRSV RNA. To examine this, we performed an m^6^A-RNA immunoprecipitation of RNA isolated from hRSV-infected Calu-3 cells treated with or without daprodustat and noted a significant reduction in viral N gene expression postinfection (*SI Appendix*, Fig. S7*F*). We observed an enrichment of m^6^A-modified hRSV-G RNAs compared to NS1 and N transcripts consistent with previous reports (69), with daprodustat treatment significantly reducing the level of m^6^A methylated hRSV-G transcripts (Fig. 7*D* and *SI Appendix*, Fig. S8*A*). Further, we confirmed that modulation of m^6^A methylation by daprodustat extended to PVM, as significantly less m^6^A-modified PVM-G transcripts were precipitated from the total lung RNA isolated from vehicle and treated animals (*SI Appendix*, Fig. S8*B*). We next evaluated whether daprodustat retains antiviral activity against hRSV in the absence of the two principal m^6^A demethylases, ALKBH5 and FTO. Individual knockdown of *ALKBH5* or *FTO* partially reduced the antiviral activity of daprodustat, while knockdown of both demethylases abolished its activity in hRSV-infected Calu-3 cells (Fig. 7*E* and *SI Appendix*, Fig. S8*C*). Daprodustat increased *ALKBH5* expression but had little impact on *FTO* transcript levels in line with previous reports describing HIF-mediated regulation of these demethylases (70). Further, knockdown of *ALKBH5* and *FTO* ablated the daprodustat-mediated increase in IFN-β promoter activity during hRSV infection (*SI Appendix*, Fig. S8*D*) indicating that their activity is necessary for the increased innate sensing of hRSV RNA. To explore this further, we generated hRSV-G transcripts by in vitro transcription (IVT) that possess no posttranscriptional modifications and thus can be classified as m^6^A-deficient. Transfection of IVT-hRSV-G RNA into IFN-β reporter cells induced significantly higher levels of the promoter activity compared to RNA derived from viral particles (*SI Appendix*, Fig. S8*E*), consistent with our earlier findings and previous reports that m^6^A methylation of hRSV RNA limits innate immune detection (69).

To functionally assess whether m^6^A modification of the viral RNA is required for the antiviral activity of daprodustat, we generated m^6^A-deficient hRSV by propagating hRSV in METTL3 and METTL14 KD cells as previously described (69). This approach produced RSV stocks with significantly reduced levels of m^6^A RNA (*SI Appendix*, Fig. S8*F*) that were used to infect Calu-3 cells with or without daprodustat treatment. Importantly, Daprodustat did not suppress replication of the m^6^A-deficient hRSV, in contrast to wild-type hRSV (Fig. 7*F*). Consistent with these findings, daprodustat treatment of the reporter cells transfected with RNA derived from the m^6^A-deficient hRSV did not induce IFN-β promoter activity (Fig. 7*G*). Taken together, these findings demonstrate that the antiviral effect of daprodustat is mediated through modulation of viral RNA m^6^A modification, promoting detection through the innate nucleic acid sensing pathway (Fig. 7*H*).

## Discussion

This study demonstrates that activation of HIF-signaling by the PHDi daprodustat limits pneumoviral replication in vivo and *in vitro,* through enhanced activation of innate sensing. Daprodustat treatment of PVM-infected mice led to a significant reduction in pulmonary viral antigen expression, coupled with a reduced inflammatory response in the lungs. There is accumulating evidence that HIFs enhance intrinsic pathways to limit damage in acute lung injury through promoting blood vessel repair and the regeneration of alveolar type II pneumocytes (71, 72). Coupled with recent findings showing that HIFs play a key role in the differentiation of type I and II alveolar cells during embryonic development (73), implicate hypoxic signaling as a key protective pathway in the lung. Recent findings have demonstrated a protective influence of Vadadustat treatment, a new generation prolyl hydroxylase inhibitor, improves clinical outcomes in patients with severe lung injury resulting from SARS-CoV-2 infection as well as enhancing airway repair in murine models of infection (31). Analysis of the murine lung transcriptome revealed that daprodustat promoted expression of numerous innate immune response genes in PVM infection, including IFN-stimulated genes such as *Isg15*. The increased *Isg15* expression in lung and spleen tissues further supports the immunomodulatory effects of daprodustat and extends our earlier report that HIF restricted nucleolin expression (44), demonstrating further roles for HIF to limit pneumovirus replication through modulation of innate sensing. Additionally, DCQ analysis of our transcriptomic datasets revealed modulation of specific immune cell subsets in the lung of daprodustat-treated animals infected with PVM. To improve the accuracy of these findings, application of either single-cell or spatial transcriptomic techniques would resolve the specific changes in the immune cell compartments induced by HIF signaling in pneumoviral infection. Using the natural mouse pathogen PVM to model pneumoviral disease allowed us to study viral replication in an in vivo setting that emulates many of the clinical features of severe hRSV infection, such as acute pneumonia, and leukocyte recruitment (57, 58). We observed that activation of HIF signaling limited both pulmonary viral antigen expression and immune cell recruitment compared with vehicle control groups. In addition, levels of *Isg15* mRNA in the spleen were significantly increased in PVM-infected mice treated with daprodustat. Protein modification by ISG15 (ISGylation) represents a significant element of the IFN-induced immune response, regulating critical antiviral factors important for the control of several viral infections such as Influenza and Sindbis virus (74, 75). Elevated expression of *ISG15* was previously reported in hRSV-infected nasopharyngeal washes and in vitro model systems (14), consistent with our observation in the lung and spleen of PVM-infected mice. ISG15 has been implicated as a posttranslational regulator of HIF-1α expression, with ISGylation suppressing HIF-mediated gene transactivation and destabilizing HIF in a proteasome-dependent manner (76). Notably, HIF-1α has been shown to induce transcriptional expression of *ISG15* through the presence of multiple hypoxic response elements in the *ISG15* promoter region (76, 77). Our transcriptomic analysis of infected PVM lungs revealed that HIF increased the expression of numerous innate immune response genes. Collectively these data suggest that in the context of pneumoviral infection, HIF acts in tandem with innate sensing to limit viral replication. This potential synergism may explain the marked increase in *Isg15* RNA expression in both the spleen and lungs of PVM-infected animals and is supported by our confocal imaging data showing nuclear ISG15 staining in daprodustat-treated hRSV-infected cells (Fig. 5*B*). Importantly, using smFISH labeling of viral RNA combined with immunofluorescent imaging of the viral N protein, daprodustat treatment limited the number of hRSV replication sites or IBs. We have previously demonstrated using this approach that activation of HIF limits the establishment of SARS-CoV-2 replication sites in early infection (42, 78). While there are significant differences in the biology and replication cycles of coronaviruses and pneumoviruses, the restriction by HIF in their respective replication complex establishment may highlight common pathways that these viruses subvert to attain productive infection. These potential shared mechanisms could enhance our knowledge of virus–host interactions and contribute to more effective prevention and treatment strategies for respiratory viral infections.

In the lung HIF-1α is ubiquitously expressed in all pulmonary cell types (79), while HIF-2α is more commonly expressed in cells of the vascular endothelium and type II pneumocytes (80). Our siRNA and HIF-overexpression models allowed us to demonstrate a role for both HIF-alpha subunits to restrict viral replication indicating redundancy between either isoform. Blocking IFN signaling abrogated the antiviral activity of daprodustat, indicating that the type I IFN pathway is required to limit viral replication, in agreement with the enhanced innate immune gene expression seen in vivo. Using ISG15 as a marker for innate gene activation, we found that ablation of HIF expression in the context of viral infection led to a reduction in ISG15 protein expression. In addition, disruption of RLR signaling significantly reduced the impact of daprodustat on both viral replication and innate immune activation. RLRs are principal PRR that detects hRSV RNA in the early stages of infection to initiate the innate antiviral response. Previous studies have shown that genetic silencing of RIG-I in vitro had little impact on hRSV growth in lung epithelial cell lines (81), in agreement with our findings that showed a modest increase in replication in RIG-I knock-out cells. However, children with loss of function mutations in the *IFIH1* gene, which encodes MDA5, show higher susceptibility to hRSV-associated bronchiolitis, due to an inability to produce IFN-β (82), highlighting the importance of RLR sensing in the context of clinical infection. We observed that activation of the IFN-β promoter in response to hRSV infection was dependent on both RIG-I and MDA5, as only cells with ablated expression of both factors or of MAVS failed to mount a response to hRSV infection, indicating redundance between RIG-I and MDA5 (Fig. 7*B*). Importantly, we demonstrated that enhancement of IFN-β promoter activity by daprodustat in hRSV-infected cells required functionality of both RIG-I and MDA5.

To dissect the mechanism by which HIF activation enhances innate immune sensing, we focused on the direct interaction between viral RNA and host PRRs. We found that viral particle-associated RNA was sensed primarily by RIG-I when transfected into cells. Daprodustat treatment of viral RNA, but not poly I:C, transfected cells resulted in increased IFN-β promoter activation, suggesting that HIF specifically promotes the detection of hRSV RNA rather than being a generic activator of RIG-I/MDA5 sensing. Importantly, neither RIG-I or MDA5 appear to be directly regulated by HIF and lack a hypoxic response element in their promoter regions. hRSV, like many RNA viruses, contains RNA modifications within its genome, the most common of which is N^6^-methyladenosine (m^6^A) (83) and there is an accumulating body of evidence suggesting that viruses acquire such modifications as a strategy to avoid host innate immunity (83, 84). In hRSV, m^6^A sites are concentrated in the G gene and depletion of these motifs results in increased immune recognition of viral RNA by RIG-I (69). In line with these findings, we noted that HIF-activation via daprodustat treatment had little impact on the replication of m^6^A-deficient hRSV, highlighting a alternative mechanism for HIF signaling to increase detection of pneumoviral RNA through modulation of RNA-methylation.

In conclusion, this study identified daprodustat as a promising host-directed antiviral agent against pneumoviruses, acting through HIF-dependent enhancement of cellular nucleic acid sensing. These findings provide important insights into the molecular interplay between hypoxia signaling and antiviral immunity.

## Methods

### Animals, PVM Infection, and Daprodustat Treatment.

Animal work was approved by the local University of Liverpool Animal Welfare and Ethical Review Body and performed under UK Home Office Project Licence PP4715265. Female BALB/c mice (BALB/cAnNCrl), 6 to 8 wk of age, were purchased from Charles River and maintained under SPF barrier conditions in individually ventilated cages. Mice were divided into two groups for treatment with vehicle or daprodustat (n = 4 per group). Animals were treated with 30 mg/kg of daprodustat (MedChem Express) by oral gavage 2 h preinfection and then twice a day until they were euthanized in accordance with the home office project licence guidelines stated above. Drug was dissolved in 99% double distilled H_2_O, 0.5% methyl cellulose, and 0.5% Tween-80. Mice were intranasally infected with the PVM J3666 strain at a dose of 10^4 PFU under light ketamine anesthesia. The PVM J3666 strain (a kind gift of Prof Andrew Easton, University of Warwick) was passaged twice in mice. Lungs were harvested, and the supernatant was titrated before the in vivo infection experiment. Animals were euthanized in accordance with the home office project licence guidelines by cervical dislocation on day 5 postinfection, and tissues were collected for RNA viral titer and histological examination.

### Quantification and Statistical Analysis.

All data are presented as mean ± SD. *P* values were determined using a Mann–Whitney test or Student’s *t* test (two-group comparisons) or a Kruskal–Wallis ANOVA (multigroup comparisons) using PRISM version 10. In the figures *P < 0.05, **P < 0.01, ***P < 0.001, and ****P < 0.0001.

See *SI Appendix* for additional *Methods*.

## Supplementary Material

Appendix 01 (PDF)

## Data Availability

All data included in this study are available through Mendeley Data, DOI: 10.17632/t82zmcbj56.1 (85).
