# Supplementary material for: Hypoxia inducible factors regulate pneumovirus replication by enhancing innate immune sensing
Source: Proc Natl Acad Sci U S A. 2026 Apr 7;123(15):e2506647123. doi: 10.1073/pnas.2506647123 (PMC13079924; doi:10.1073/pnas.2506647123)
Supplement: Supplementary file 1 — Appendix 01 (PDF) [file pnas.2506647123.sapp.pdf]

PNAS

Supporting Information for

Hypoxia inducible factors regulate Pneumovirus replication by enhancing innate immune sensing

Authors: Jiyeon Ha[1]*, Parul Sharma [2]*, Sammi Ta[3], Senko Tsukuda[3], James M. Harris[3], Rebekah Penrice-Randal[2], Eleanor Bentley[2], Adam Kirby[2], Daniele F. Mega[2], David A. Matthews[4], Peter Balfe[3], Jan Rehwinkel[5], Anja Kipar[2,6+], James P. Stewart[2+], Jane A. McKeating[1,3+]✉, Peter A.C. Wing[1+]✉

* Corresponding authors: Jane A McKeating, Peter A.C. Wing.
Email: jane.mckeating@ndm.ox.ac.uk, peter.wing@ndm.ox.ac.uk

This PDF file includes:

Figures S1 to S8
Legends for Figures S1 to S8
SI References

Other supporting materials for this manuscript include the following:

Supplementary Methods

Supplementary Figures

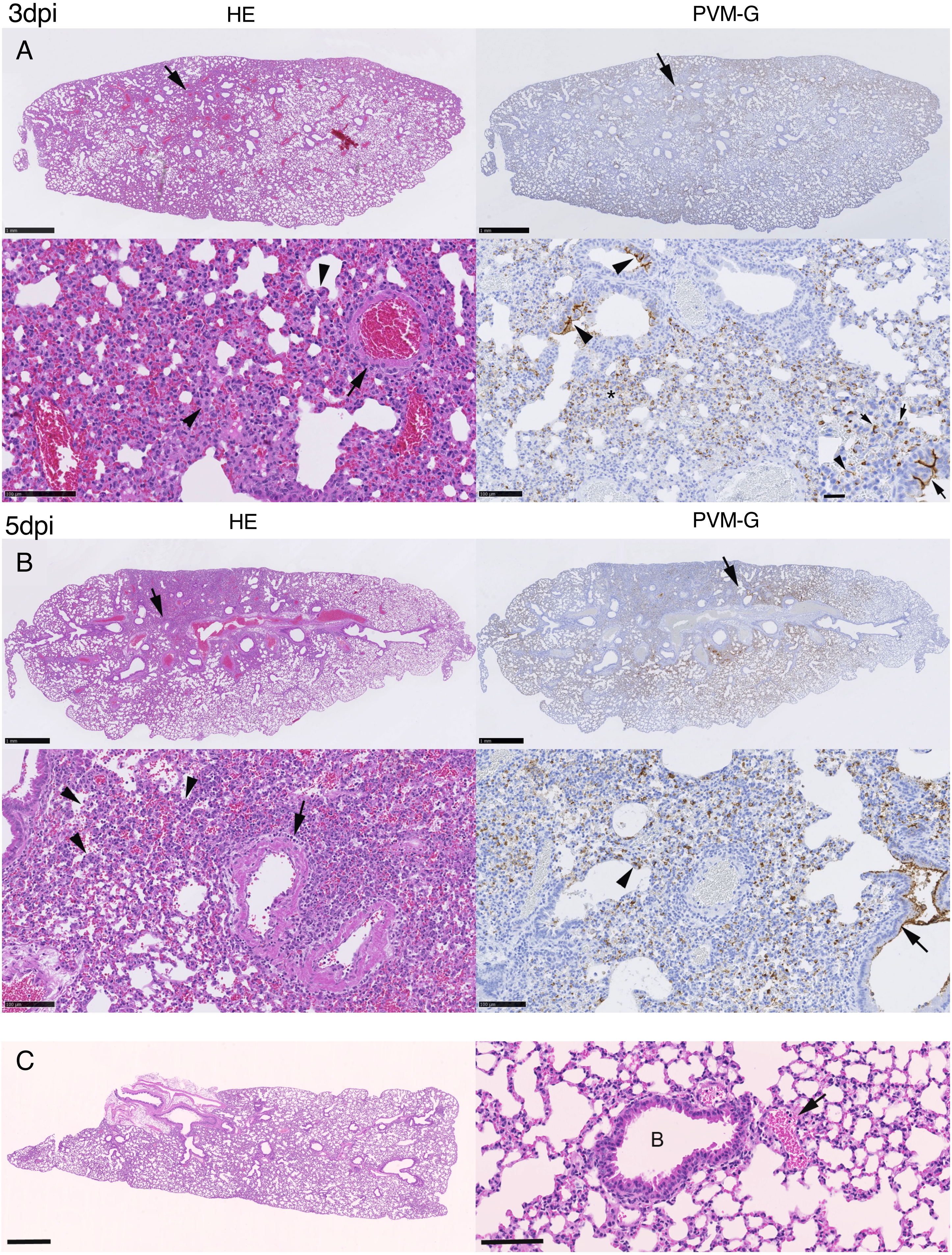

Supplementary Figure 1

Supplementary Figure 1: ***Pulmonary histopathology of PVM infected mice***

Histological features and viral antigen expression were assessed at 3 and 5 dpi. **(A)** At 3 dpi, the lung parenchyma showed focal areas of increased cellularity (overview: arrow). Arrowheads of the higher magnification image show mild increases in interstitial cellularity and large activated type II pneumocytes in HE stained sections. Consecutive sections were stained for PVM-G that showed widespread expression (right image - overview). Large arrowheads on the bottom inset image indicate abundant infected type I pneumocytes, small arrowheads show infected type II pneumocytes in an area of increased cellularity (asterisk). The inset image bottom right, shows PVM-G expression in patches of respiratory epithelial cells, mainly along the luminal cell borders (arrowheads)**. (B)** At 5 dpi, focal dense areas of the parenchyma are indicated by the arrow on the overview images (HE stains). Arrowheads on the bottom left higher magnification image indicate the presence of desquamed alveolar macrophages/type II pneumocytes and some leukocytes in alveolar lumina and mild leukocyte recruitment and perivascular accumulation (arrow). Viral infection is widespread, with abundant viral antigen expression in alveoli (type I and II pneumocytes; arrowhead) and in respiratory epithelial cells in a few bronchioles. The abundant unstained cells are infiltrating leukocytes. The arrow in the overview image highlights the area depicted in the higher magnification. **(C)** Lung, mock infected mouse. The parenchyma is unaltered, as shown in the overview (left) and in a closer view of a bronchiole (B) with adjacent vessel (arrow), surrounded by unaltered alveoli. Representative images are shown, and scale bars represent 50μm.

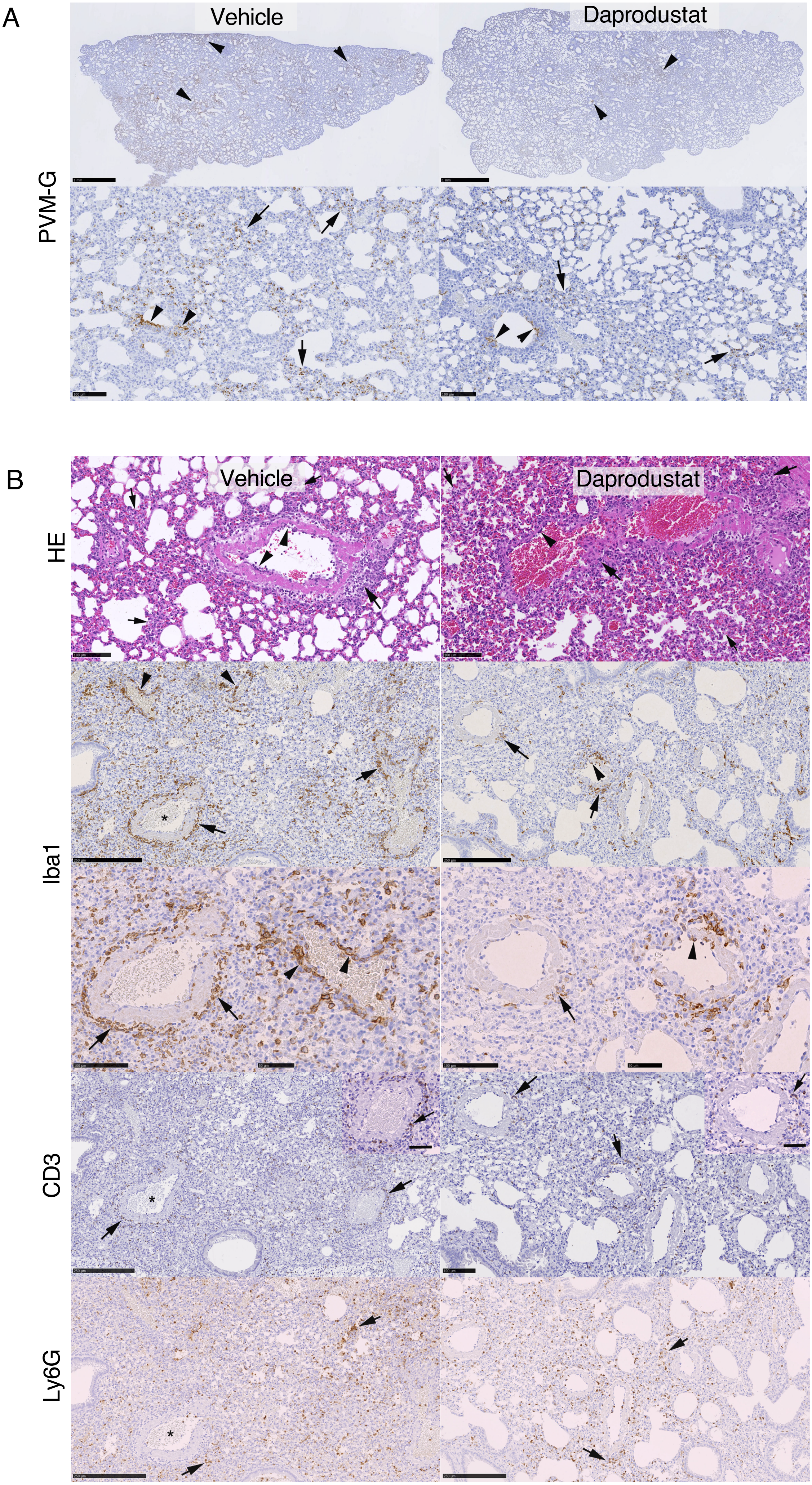

Supplementary Figure 2

Supplementary Figure 2: Histological features of Daprodustat treated PVM infected mice.

(A) Immunohistology of viral antigen (PVM-G) in longitudinal sections of the left lobe of the lung at 3dpi. Arrowheads indicate areas of widespread viral antigen expression in the overview images (top row). Arrowheads in the higher magnification images (bottom row) highlight viral antigen expression in bronchiolar respiratory epithelial cells and arrows indicate expression in large groups of alveoli with abundant infected type I and II pneumocytes. **(B)** Immunohistology of longitudinal sections of the lung at 5 dpi of a vehicle treated (left column) and Daprodustat treated animal (right column). Arrowheads in the HE stained sections (top layer) identify muscular veins with leukocytes rolling along the endothelial cells, showing evidence of leukocyte recruitment into the tissue. Large arrows indicate examples of perivascular accumulation of mononuclear cells and small arrows alveoli with desquamed alveolar epithelial cells/alveolar macrophages in the lumen. Consecutive sections were stained for the monocyte/macrophage marker Iba1 (second and third layer). Arrows indicate areas of perivascular infiltrates and arrowheads show areas where monocytes/macrophage are emigrating from and accumulating around vessels. Macrophages are far less abundant in the lungs of Daprodustat treated mice where they are also present in the minimal perivascular infiltrates (arrows) accompanied by evidence of monocyte rolling (arrowhead). T cells (CD3+: bottom layer) are present in the lungs of both groups of mice in low numbers. The arrows point at T cells in perivascular infiltrates (insets: higher magnification). Neutrophils (Ly6G+; bottom layer) are present in the lungs of both groups of mice in low numbers. They are mainly seen in the focal areas of alveolar damage and inflammatory infiltration (arrows). Representative images are shown, and scale bars represent 50μm.

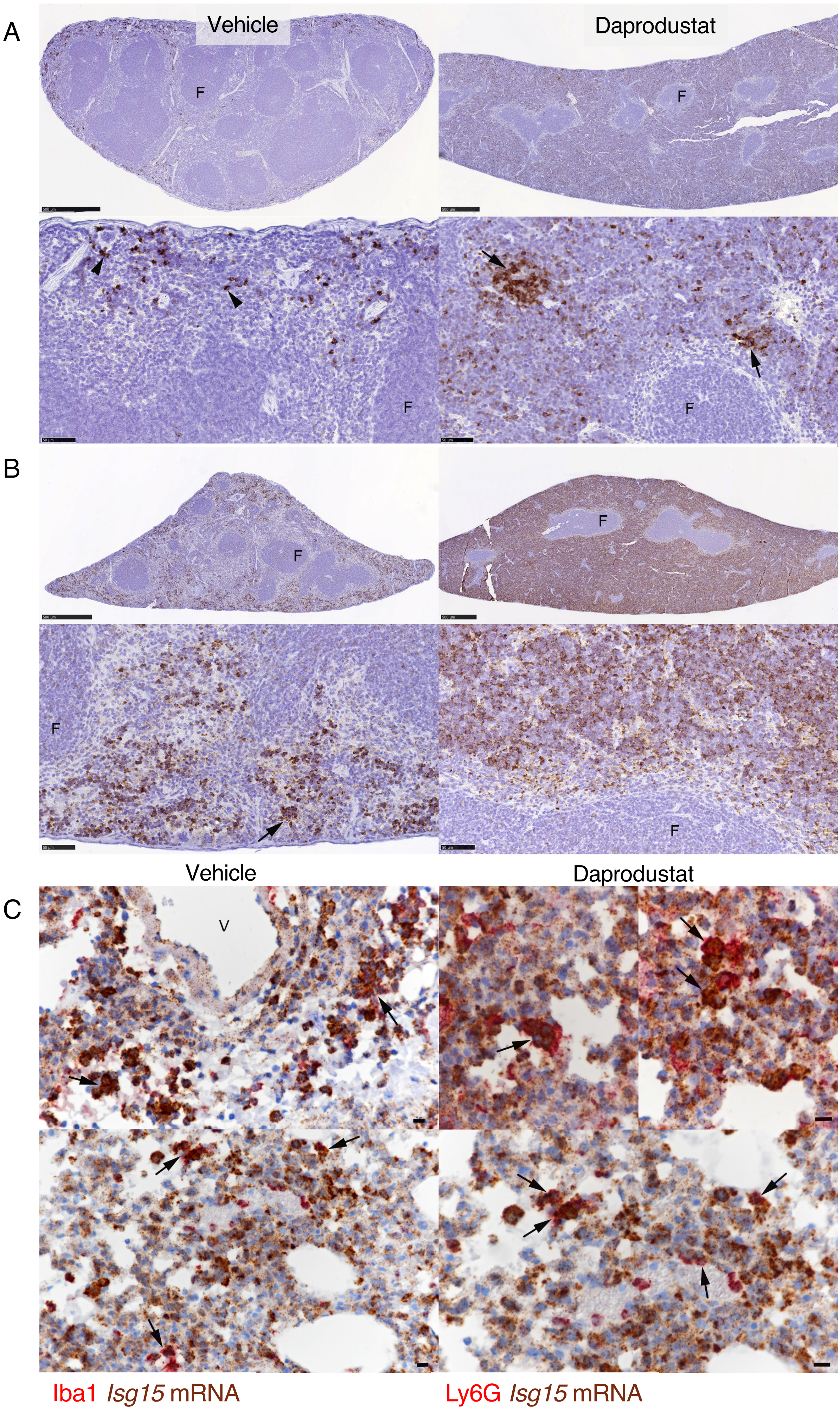

Supplementary Figure 3

Supplementary Figure 3: ISG15 gene expression in the spleen and infiltrating leukocytes in the lung after PVM infection.

(A) Detection of *Isg15* mRNA expressing cells in the spleen of untreated (left column) and Daprodustat treated (right column) mock infected mice (top layers). In untreated mock infected mice, individual *Isg15* mRNA positive cells are present in the red pulp (arrowheads). In Daprodustat treated mock infected mice, where the red pulp is of much higher cellularity, numerous *Isg15* mRNA positive cells are observed. These form variably sized aggregates (arrows). **(B)** In untreated mice after PVM infection, the number of *Isg15* mRNA positive cells is substantially increased. Again, these positive cells form aggregates (arrow). RNA-ISH, with DAB as chromogen and haematoxylin counterstain. F: follicle. Bars = 25 µm in higher magnifications. **(C)** Lungs from untreated animals after PVM infection (5 dpi). Identification of *Isg15* transcripts in infiltrating leukocytes. Macrophages (Iba1+) with *Isg15* mRNA signal are abundant in a perivascular infiltrate (left: arrows). A higher magnification identifies *Isg15* mRNA in Iba1 positive alveolar macrophages and recruited, infiltrating macrophages (right: arrows). Neutrophils (Ly6G+) with *Isg15* mRNA signal are present in the parenchymal inflammatory infiltrates (left: arrows). The higher magnification highlights the nuclear morphology of the *Isg15* mRNA positive neutrophils, confirming that Ly6G co-expressing cells are neutrophils (right: arrows). *Isg15* RNA-ISH, with DAB as chromogen, followed by immunohistology with AEC as chromogen, haematoxylin counterstain V: vessel. Representative images are shown, and scale bars represent 10µm.

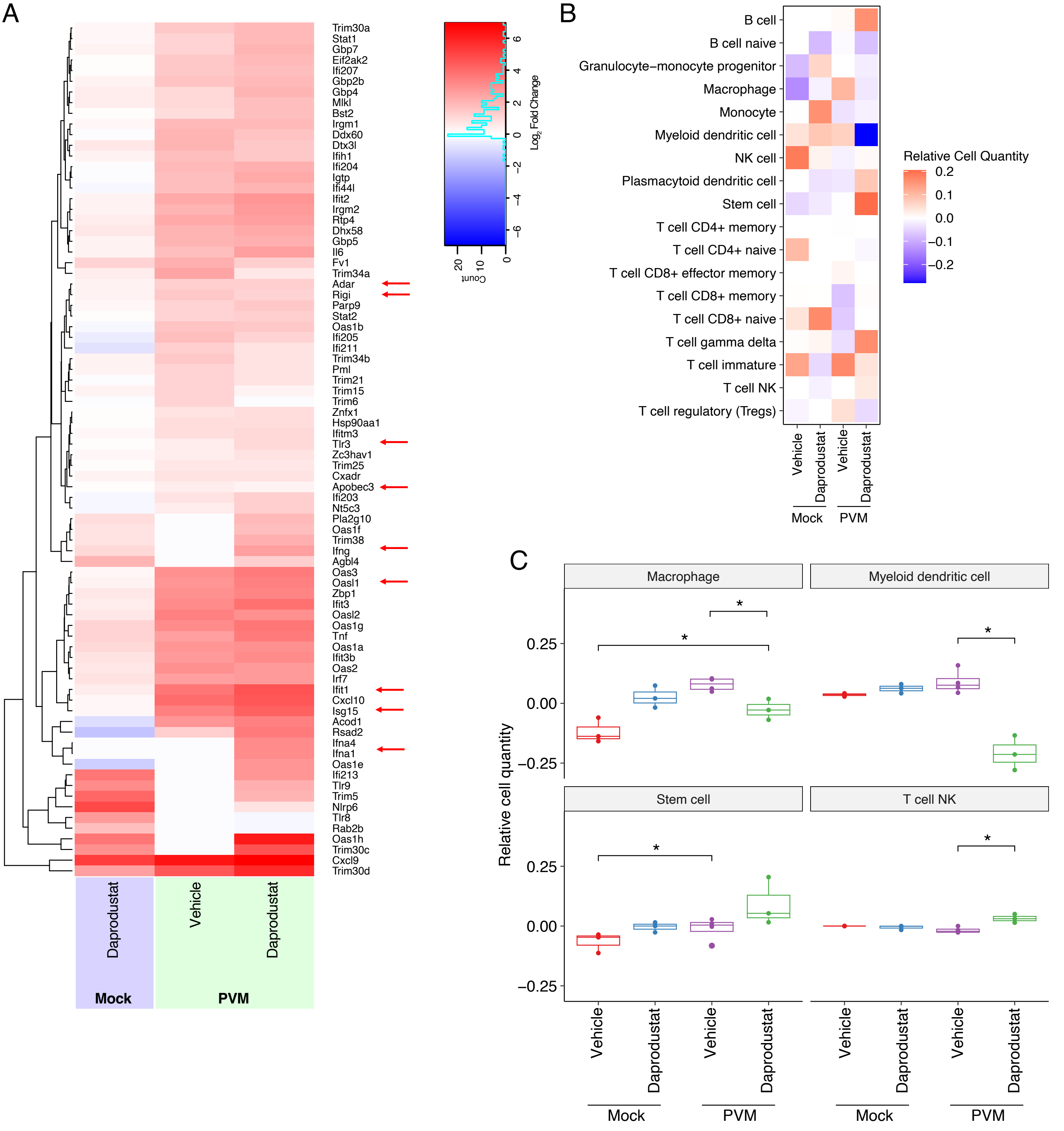

Supplementary Figure 4

Supplementary Figure 4: The impact of Daprodustat treatment on innate immune gene expression in uninfected and PVM infected mice.

(A) Hierarchical clustering of innate response genes in murine pulmonary transcriptomes either treated with Daprodustat or infected with PVM at 3dpi and treated with either vehicle or Daprodustat compared to uninfected animals. Arrow denotes key innate immune genes and colours are derived from the $\log_2$ fold change in expression relative to uninfected, untreated animals. **(B)** Heatmap showing the average relative abundance of immune cell types estimated by Digital Cell Quantification (DCQ) from normalised gene counts using the immunedeconv package. Cell types are shown on the y-axis and experimental groups are on the x-axis. Values represent the mean relative cell quantity per group. **(C)** Boxplots showing the distribution of relative abundance for a given cell type across four experimental groups: uninfected and PVM infected mice treated with or without Daprodustat. Statistical differences were assessed using Kruskal–Wallis tests followed by Dunn's post hoc tests with Benjamini–Hochberg correction. Asterisks indicate significant pairwise comparisons (*$p < 0.05$, **$p < 0.01$).

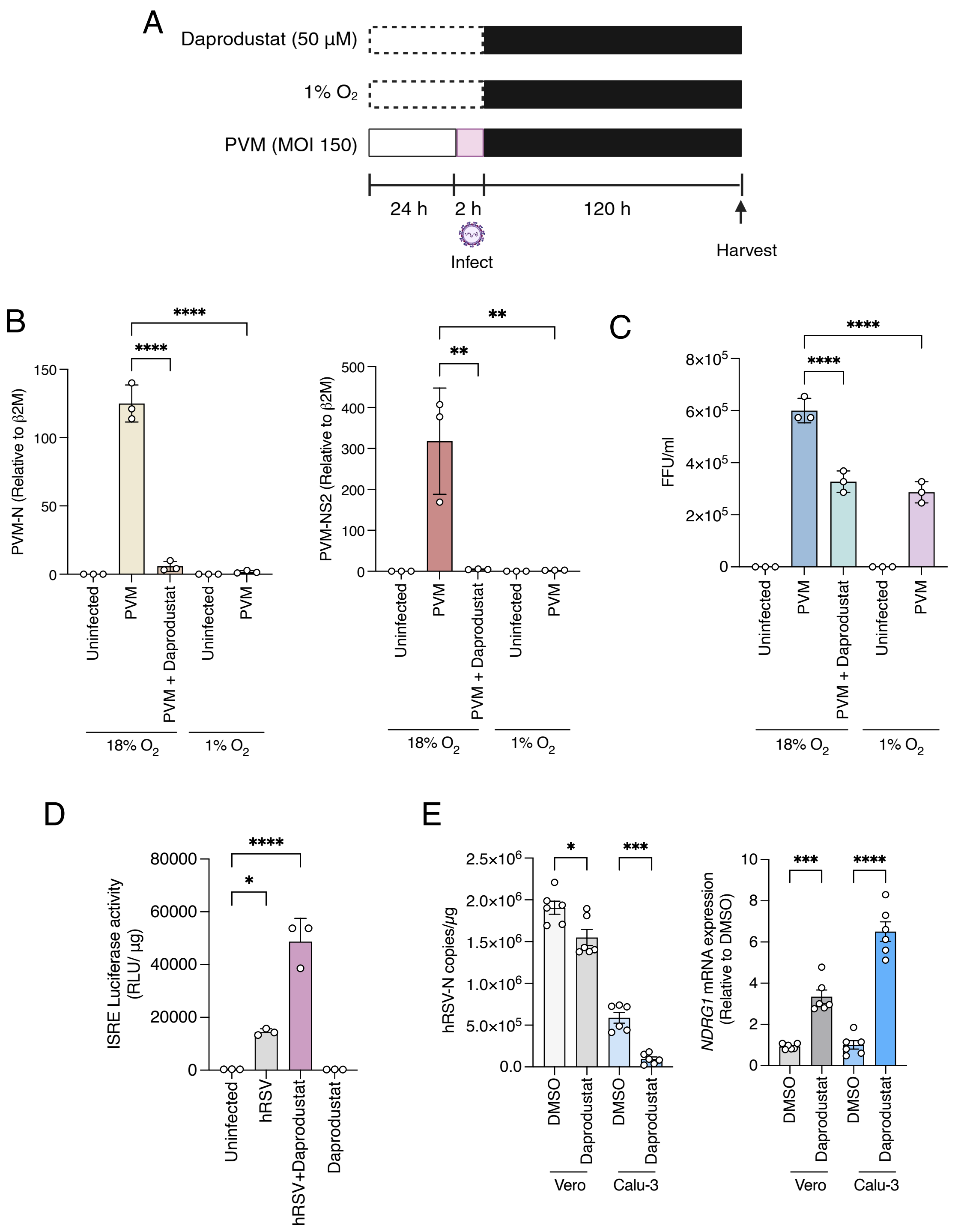

Supplementary Figure 5

Supplementary Figure 5: Daprodustat restricts PVM replication in vitro and induces innate sensing activity during hRSV infection.

(A) Experimental schematic of PVM infection of BHK cells, where cells were infected at an MOI of 150 for 2h followed by removal of the inoculum. Cells were subsequently incubated in either 1% O_2 or treated with 50µM of Daprodustat for 5 days. **(B)** Quantification of PVM N and NS2 transcripts by RT-qPCR, expressed relative to the uninfected control. Data is from three biological replicates and is plotted as mean ± SD. Statistical significance was determined by ANOVA; p<0.01 = **, p<0.0001 = ****. **(C)** At 5 dpi, PVM Infectious titre was assessed by FFU assay using an antibody against PVM-G. Data is representative of triplicate biological replicates and plotted as mean ± SD. Statistical significance was determined by ANOVA, p<0.01 = **, p<0.0001 = ****. **(D)** HEK293-ISRE-reporter cells were treated with supernatants from hRSV (MOI 1) infected HEp-2 cells with or without Daprodustat (50 µM). At 24 hpi, cell lysates were measured with Luciferase activity. **(E)** Vero or Calu-3 cells were infected with hRSV (MOI1) and treated with 50µM Daprodustat for 48h and hRSV-N or NDRG1 expression mRNA measured by RT-qPCR (mean ± SEM, n = 6, ANOVA, p<0.05 = *, p<0.001 = ***.)

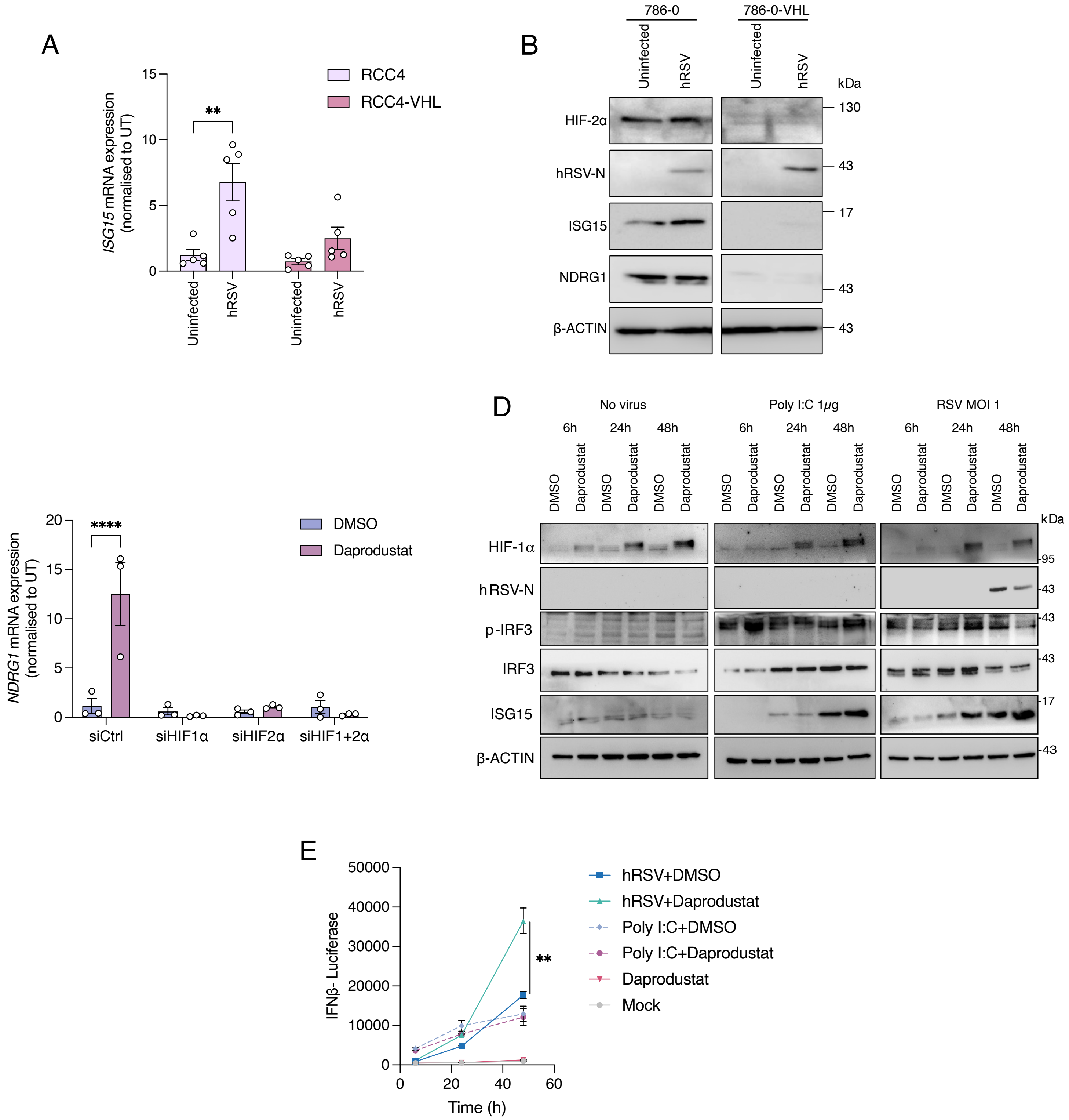

Supplementary Figure 6

Supplementary Figure 6: ISG15 expression is HIF-dependent.

(A) *ISG15* gene expression in mock and hRSV infected RCC4 and RCC4-VHL cells. **(B)** 786-0 and 786-0-VHL cells were infected with hRSV for 48h (MOI 1) and cell lysates probed for hRSV-N, ISG15, HIF-2α, and NDRG1 expression by immunoblot. **(C)** *NDRG1* gene expression in Calu-3 cells transfected with siRNAs against HIF-1 α and HIF-2α. **(D)** Expression of IRF3, pIRF3, ISG15, HIF-1α and hRSV-N was assessed in Calu-3 cells treated with stimulated with Poly-I:C or infected with hRSV (MOI 1) followed by treatment with Daprodustat 2h post infection or treatment. Cells were sampled at 6, 24 and 48h post treatment and expression of indicated proteins assessed by western blot. **(E)** HEK293-IFN-β luciferase reporter cells were infected with hRSV at an MOI of 1 followed by treatment with 50μM of Daprodustat. Luciferase reporter activity was quantified at the indicated timepoints over the course of 48h from both infected and uninfected cells treated with DMSO or Daprodustat.

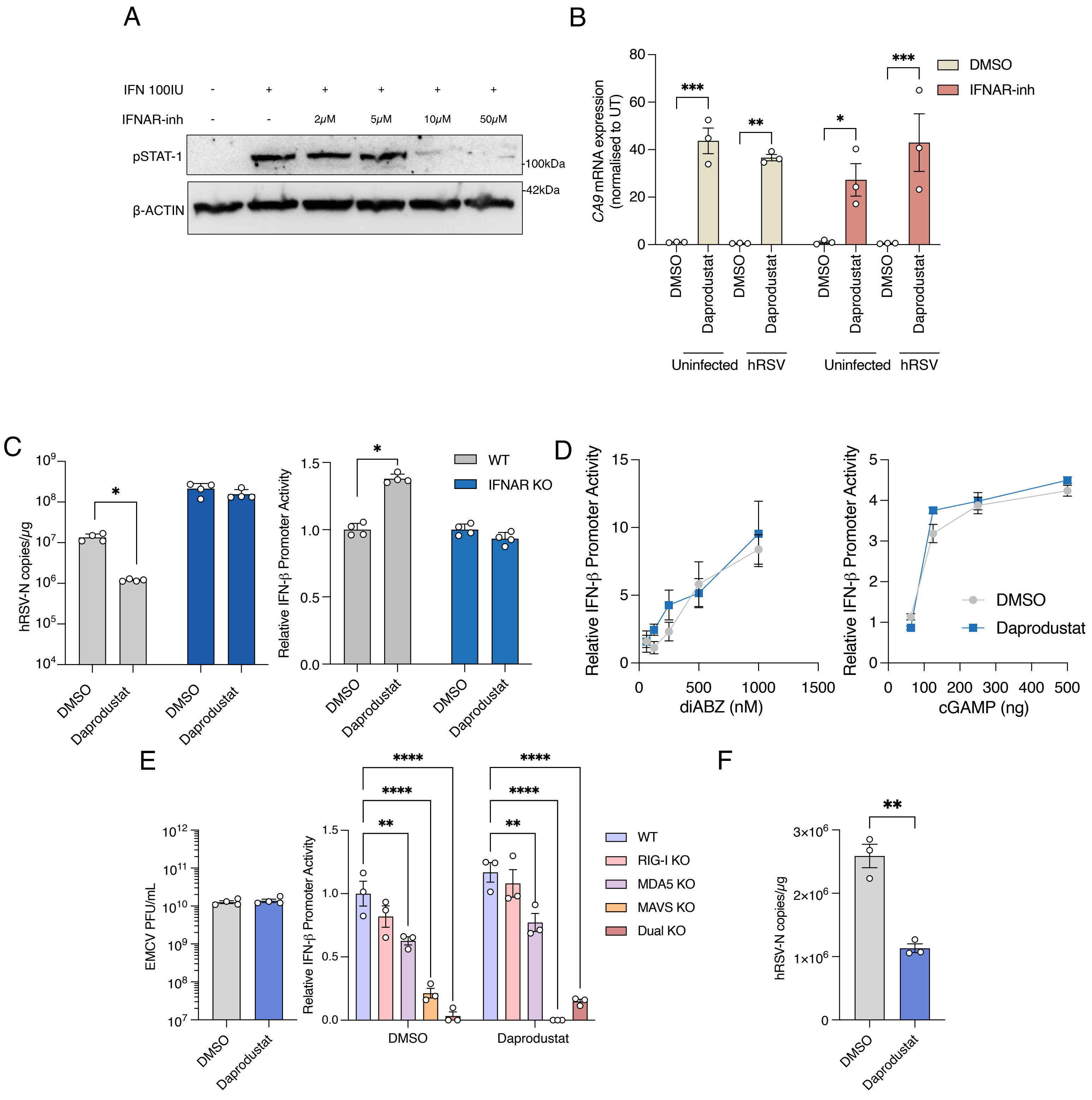

Supplementary Figure 7

Supplementary Figure 7: The antiviral effect of Daprodustat is mediated through innate sensing.

(A) Phosphorylated STAT-1 expression in Calu-3 cells treated with IFNa (100U) and increasing dose of IFNAR-inhibitor. **(B)** *CA9* gene expression in mock and hRSV infected Calu-3 cells treated with or without Daprodustat (50µM) plus the INFAR-inhibitor (10µM) for 48h. **(C)** HEK-293 WT or IFNAR knock out cells were infected with hRSV at an MOI of 1 followed by treatment with 50µM of Daprodustat for 48h. Viral replication was assessed through quantification of hRSV-N transcripts. Supernatants from this experiment were used to treat HEK293-IFN-β luciferase reporter cells with reporter activity expressed as relative to the DMSO control. **(D)** HEK293-IFN-β luciferase reporter cells were treated with increasing doses of diABZ or cGAMP with or without 50µM of Daprodustat. Luciferase activity was assessed 48h post-treatment and data expressed as relative to the DMSO control **(E)** Quantification of infectious EMCV from 293 cells treated with or without Daprodustat. Measurement of IFN-β luciferase from WT and indicated KO cells, infected with EMCV treated with or without 50µM of Daprodustat. Data is expressed as relative to WT cells treated with DMSO **(F)** qPCR quantification of hRSV copies from infected Calu-3 cells treated with or without Daprodustat.

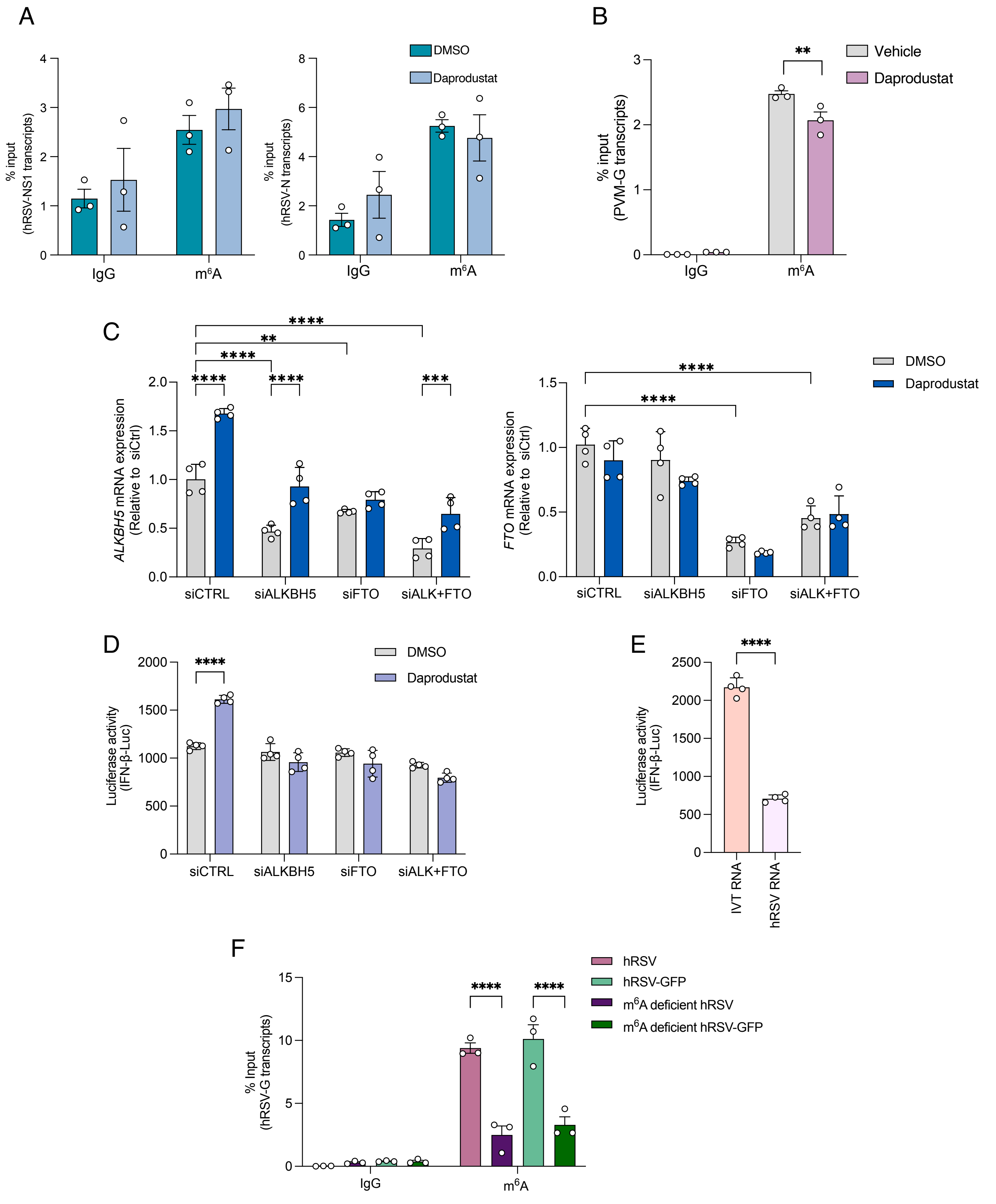

Supplementary Figure 8

Supplementary Figure 8: The antiviral effect of Daprodustat is mediated through m^{6}A RNA methylation.

(A) Quantification of methylated hRSV N and NS1 RNA transcripts from m^{6}A immunoprecipitation from total cellular RNA. **(B)** Quantification of PVM-G transcripts from m^{6}A RNA immunoprecipitation from PVM infected lung tissue respectively, expressed as % input of total RNA. RNA input was normalised so an equal number of viral copies was used in the pull-down. **(C)** mRNA expression of *ALKBH5* and *FTO* in Calu-3 cells transfected with siRNAs targeting both genes. **(D)** HEK293-IFN-β-promoter luciferase cells were treated with supernatants from hRSV (MOI 1) infected Calu-3 cells transfected siRNA targeting *ALKBH5*, *FTO* or both with or without Daprodustat (50 μM). Luciferase activity was quantified 24h post treatment. **(E)** *In vitro*–transcribed hRSV-G transcripts or RNA extracted from hRSV viral stocks was transfected into HEK293-IFN-β-promoter luciferase cells followed by quantification of luciferase activity. **(F)** Quantification of hRSV G transcripts by m^{6}A RNA immunoprecipitation from RNA isolated from wild-type and GFP- hRSV.

Supplementary Methods

Histological and immunohistological examination and RNA-ISH

The left lungs and spleen were fixed in 10% buffered formalin for 48 h, and stored in 70% ethanol until processing. Lungs (longitudinal section) and spleens (cross sections) were trimmed and embedded in paraffin wax. Consecutive sections (3 µm) were prepared and stained with haematoxylin and eosin (HE) for histological assessment and subjected to immunohistology (IH) for the detection of PVM-G antigen. IH was performed using the horseradish peroxidase (HRP) method. Briefly, after deparaffination, sections underwent antigen retrieval in citrate buffer (pH 6) for 20 min at 98°C, followed by incubation with mouse anti-PVM-G diluted in dilution buffer (Agilent Dako) overnight at 4°C. After blocking of endogenous peroxidase (peroxidase block, Agilent Dako) for 10 min at room temperature (RT), incubation) samples were incubated with rabbit anti-mouse IgG (Abcam) for 1 h at RT, followed by rabbit-HRP (Envision+System) for 30 min at RT in an autostainer (Agilent Dako). Sections were counterstained with haematoxylin. Further sections were stained for Iba1 (monocytes/macrophage marker) and CD3 (T cell marker) as previously described(1). To detect *ISG15* mRNA expression, RNA-ISH was performed using the RNAscope ISH method (Advanced Cell Diagnostics (ACD), Newark, California), Mus musculus ISG15 ubiquitin-like modifier (Isg15) (Mm-Isg15-O1; cross-detects Gm9706) oligoprobes, and the automated RNAscope 2.5 Detection Reagent Kit (brown) according to the manufacturer's protocol, and as previously described(2). To determine whether monocytes/macrophages and neutrophils express *ISG15* mRNA, a combined RNA-ISH/IHC protocol was applied after established immunohistology protocols for Iba and Ly6G (neutrophil marker) proved to maintain immunolabeling after the ISH pretreatment steps (baking, deparaffinizing, incubating in RNAscope Hydrogen Peroxide, cooking in RNAscope 1X Target Retrieval Reagents solution, and incubating in RNAscope Protease Plus). Sections were then subject to the RNA-ISH protocol, followed by staining for Iba1 and Ly6G, respectively, combining previously published protocols(1, 2).

hRSV and PVM in vitro propagation

Human hRSV subtype A (A2 strain) was grown in HEp-2 cells and cell lysates concentrated using Vivaspin® 20, 10,000 MWCO PES columns (Sartorius) as reported previously(3). Briefly, cells were infected at MOI 0.2 for 2h, the inoculum removed, cells washed with PBS and cultured in DMEM containing 5% FBS. After 5 days, cells were harvested and cellular lysates clarified and concentrated using a Vivaspin® 20 column. GFP-hRSV was propagated using the same method as described for hRSV(3). BHK-21 cells were cultured in DMEM containing 10% FBS and infected with PVM (J3666 strain) at MOI 0.0025 at 33 °C in 5% CO_2. After 10 days, the cell lysates concentrated using 10,000 MWCO PES columns (Sartorius).

The hRSV, GFP-hRSV and PVM viral stocks were aliquoted, snap frozen in liquid nitrogen and stored at -80ºC. To generate m⁶A-deficient hRSV, HEp-2 cells were co-transfected with siRNAs targeting METTL3 and METTL14 using DharmaFect 4 (Thermo Fisher). After 24 h, cells were infected with hRSV or GFP-hRSV under the same conditions used for WT virus propagation, and viral lysates were collected and processed identically to yield m⁶A-deficient viral stocks.

Cells and Reagents

All cells, including co-cultures, were cultured at 37°C and 5% CO_2 in a standard culture incubator and exposed to hypoxia using an atmosphere-regulated workstation set to 37ºC, 5% CO_2 and 1% O_2 (Invivo i200, Baker-Ruskinn Technologies). Calu-3 cells were cultured in Advanced DMEM (Sigma-Aldrich) supplemented with 10% fetal bovine serum (FBS), 2mM L-glutamine, 100 U/mL penicillin and 10 mg/mL streptomycin (Invitrogen). HEp-2, Vero, RCC4, RCC4-VHL, 786-0, 786-0-VHL, BHK-21, HEK293-ISRE-Luc reporter, HEK293-P125 (IFNβ-Luc promoter cell) WT, RIG-I, MDA5, RIG-I/MDA5 KO and MAVS KO cells were cultured in DMEM (Sigma-Aldrich) with the same supplements as described above (see **Supp Table 1**). Human PBECs were purchased from Lifeline Cell Technologies. Airway epithelial cells were cultured in Airway Epithelial Cell medium (PromoCell, Heidelberg, Germany) in submerged culture. PBECs were cultured on PureCol-coated 0.4 μm pore polyester membrane permeable inserts (Corning) in serum-free airway epithelial cell media, brought to air-liquid interface, replacing basal media with Air-Liquid Interface Epithelial Differentiation Medium (Lifeline Cell Technologies). The media was exchanged every 2 days and apical surfaces washed with PBS weekly to disperse accumulated mucus, for a minimum of 6 weeks.

RNA-seq analysis

Right upper lung lobes from PVM infected mice at 3dpi were homogenised in 1 ml of Trizol reagent (Thermofisher) using a QT tissue lyser and stainless-steel beads (Qiagen) at 50 oscillations for 5 minutes. The homogenates were clarified by centrifugation at 12,000xg for 5 min before full RNA extraction was carried out according to manufacturer's instructions. RNA was quantified and quality assessed using a Nanodrop (Thermofisher) before a total of 1μg was DNase treated using the TURBO DNA-free™ Kit (Thermofisher) as per manufacturer's instructions. RNA was sequenced using a 300bp paired-end Illumina sequencing protocol (Novogene, UK). Host reads were mapped to the human transcriptome and differential expression analysis performed using DESeq2. Pathway analyses were performed using the enrichGO package in Bioconductor using R-Studio version 2024.12.0. Relative cell quantities were estimated using Digital Cell Quantification (DCQ)(4) implemented in the immunedeconv R package (v2.1.0). Briefly, gene counts were normalised using the DESeq2 package (v1.42.1) and input into immunedeconv with the function deconvolute_mouse

(normalised_counts, "dcq"). Statistical differences across experimental groups were assessed using a Kruskal–Wallis test followed by Dunn's post hoc test for pairwise comparisons, implemented via the rstatix package (v0.7.2). Results were visualised using ggplot2 (v3.5.2). Data is available through the following accession code GSE290159.

RNA m^6A immunoprecipitation

Total RNA was extracted from hRSV (MOI 1) infected Calu-3 cells treated with or without Daprodustat using the RNeasy Mini Kit (QIAGEN). The RNA was subsequently treated with TURBO DNase (Thermo Fisher Scientific) and further purified using the RNeasy Mini Kit. RNA concentration was measured, and equal copy numbers of hRSV RNA were incubated with Protein G Agarose beads (Cell Signaling) pre-bound and either Mouse IgG (SantaCruz) or anti-m^6A Monoclonal antibody (Proteintech) in MeRIP buffer (50 mM Tris-HCl (pH 8.0), 150 mM NaCl, 0.1% NP-40, 1 mM EDTA) supplemented with RNase inhibitor (Promega). m^6A-modified RNA was eluted using 6.7 mM m^6A sodium salt, purified using Qiagen RNA extraction kit and performed RT-qPCR analysis.

Confocal immunofluorescence microscopy and smFISH

Infected cells were fixed with 4% paraformaldehyde (PFA; Sigma) in PBS for 15 min, blocked with 20mM glycine in PBS and permeabilized with 0.5% Triton X-100 in PBS for 5 min. Samples were incubated with anti-hRSV-F primary antibody for 1h at room temperature, washed and incubated with Alexa Fluor secondary antibodies (Life Technologies) for a further hour at room temperature. After washing, slides were mounted with Fluoromount G (SouthernBiotech) containing 4′,6-diamidino-2-phenylindole (DAPI) for nuclei staining. smFISH was carried out as previously reported(5). Briefly, HEp-2 cells grown on #1.5 round-glass coverslips in a 24-well plate were fixed in 4% paraformaldehyde (ThermoFisher) for 30min at room temperature. Cells were permeabilised in PBS/0.1% Triton X-100 for 10min at room temperature, followed by washes in PBS and 2X SSC. Cells were pre-hybridised in pre-warmed (37°C) wash solution (2×SSC, 10% formamide) twice for 20min at 37˚C. smFISH probes designed against the hRSV-N and P transcripts were diluted to 500nM in hybridisation solution (2×SSC, 10% formamide,10% dextran sulphate) and incubated overnight at 37˚C. Coverslips were then washed for 20min in pre-warmed wash solution at 37°C followed by counterstaining with DAPI (1µg/mL) diluted in wash solution. Cells were washed once with wash solution for 20min at 37°C and twice with 2XSSC for 10min at room temperature. Cells were imaged on an Olympus SoRA spinning disc confocal microscope. Quantification was performed using ImageJ software (Fiji). The average intensity of hRSV-RNA smFISH signal was defined based on an automatic threshold with a Minimum Error algorithm. The number of cells was quantified based on the DAPI signal. A watershed step was performed to separate nuclei in close proximity.

Plaque assays

To quantify infectious hRSV, samples from infected cells and lung homogenates from infected mice were serially diluted 1:10 and used to inoculate monolayers of HEp-2 cells for 2h. Inocula were then replaced with DMEM containing 1% FCS with a semi-solid overlay of 1.5% carboxymethyl cellulose (Sigma-Aldrich). Cells were incubated for 4-5 days, fixed in 4% PFA, stained with 0.2% crystal violet (w/v) and plaques enumerated, where the limit of detection of this assay was 10 focus forming units PFU/mL. PVM quantification in BHK-21 cells was conducted using a modified version of the standard immunofluorescent plaque assay described by Watkiss et al. (2013) (6). Lung samples from in-vivo infections were serially diluted tenfold in DMEM medium, incubated for 72 h at 33°C with 5% CO_2, and subsequently fixed in ice cold methanol and acetone and stained with PVM-G monoclonal antibody at room temperature. Plaques were visualized and counted using a fluorescent microscope (Zeiss).

Immunoblotting

Cell lysates were prepared by washing cells with phosphate buffered saline (PBS), then lysing in RIPA lysis buffer (20 mM Tris, pH 7.5, 2mM EDTA,150 mM NaCl, 1% NP40, 0.1% SDS and 1% sodium deoxycholate) supplemented with Complete TM protease inhibitor cocktail (Roche) at 4ºC for 5 min, followed by clarification by centrifugation (3 min, 12,000 rpm). Supernatants were mixed with Laemmli sample buffer, and boiled at 100ºC, separated by SDS-PAGE and proteins transferred to polyvinylidene difluoride membrane (Immobilon-P, Millipore). Membranes were blocked in 5% milk in PBS/ 0.1% Tween-20, incubated with anti-HIF-1α (BD Transduction Laboratories, clone#610959), anti-NDRG1 (Cell signalling, clone#5196) or anti-β-actin (Sigma, clone#A5441) primary antibodies and appropriate HRP-conjugated secondary Mouse (DAKO, clone#P0447) or Rabbit antibodies (Cytiva, clone#NA934V). Chemiluminescence substrate (West Dura, 34076, Thermo Fisher Scientific) was used to visualize proteins using a ChemiDoc XRS+ imaging system (BioRad). Densitometric analysis was performed using FIJI (NIH).

ISRE and IFN-β Luciferase assay

HEK293-ISRE-Luc reporter cells were infected with hRSV at an MOI of 1 for 2h, the viral inoculum was removed and the cells cultured with growth media including Daprodustat (50μM). 24h post-infection, luciferase expression was assessed using a Firefly luciferase assay system (Promega, E1500) according to the manufacturer's instructions. HEK293-p125 WT, RIG-I, MDA5, MAVS, RIG-I/MDA5 KO cell lines were seeded into 96 well plates and transfected with 150ng of viral RNA or poly I:C (Merck, P1530, Polyinosinic–polycytidylic acid sodium salt) complexed with Fugene 6 (Promega) per well. 24h after transfection, Daprodustat (50μM) was added to each well for 48h. Luciferase activity was determined as described above. For extraction of hRSV particle associated RNA, hRSV stocks were treated with

RNAse I (Thermo) to remove any non-particle associated RNA. RNase was inactivated by heating at 95ºC for 10mins and RNA extracted using TRIzol (Ambion) according to manufacturer’s instructions. Post-extraction RNA was quantified using a Nanodrop 2000 spectrophotometer (Thermo Scientific) and 150ng transfected into HEK293-P125 cells as described above.

siRNA silencing.

Scramble, HIF-1α, HIF-2α, ALKBH5, and FTO siRNAs, as well as a combined siRNA treatment, were transfected into Calu-3 cells individually or in combination using DharmaFect 4 (Thermo Fisher). At 24 h post-transfection, cells were inoculated with hRSV or hRSV-GFP (MOI 1) and treated with DMSO or 50 µM Daprodustat for 48 h. Cells were collected at 48 hpi for RT-qPCR analysis.

In-vitro transcription

In vitro transcription of RSV-G was performed using the T7-Megascript kit (LifeTech). Each 20µl reaction contained 2µl of ATP, GTP, CTP UTP, 2µL of 10X reaction buffer, 1µg of linearised template DNA and water to make up the volume to 20µl. Reactions were incubated at 37ºC for 3hrs and RNA purified using the Qiagen RNeasy kit.

RT-qPCR

RNA was extracted from infected cells using the RNeasy Mini kit (QIAGEN), according to the manufacturer’s instructions. 500 ng of RNA were used to generate cDNA using an UltraScript cDNA synthesis kit (PCR Biosystems) and mRNA expression measured using the SyGreen Blue, SYBR green qPCR kit (PCR Biosystems). For quantification ΔC_t values were defined as the difference between the target gene Ct value and the Ct value of a housekeeper gene.

Supplementary References

1. F. Seehusen *et al.*, Neuroinvasion and Neurotropism by SARS-CoV-2 Variants in the K18-hACE2 Mouse. *Viruses* **14** (2022).
2. R. Fabian *et al.*, The golden Syrian hamster (Mesocricetus auratus) as a model to decipher relevant pathogenic aspects of sheep-associated malignant catarrhal fever. *Vet Pathol* 10.1177/03009858251315115, 3009858251315115 (2025).
3. X. Zhuang *et al.*, Hypoxia inducible factors inhibit respiratory syncytial virus infection by modulation of nucleolin expression. *BioRxiv* 10.1101/2023.10.31.564923 (2023).
4. Z. Altboum *et al.*, Digital cell quantification identifies global immune cell dynamics during influenza infection. *Mol Syst Biol* **10**, 720 (2014).
5. J. Y. Lee *et al.*, Absolute quantitation of individual SARS-CoV-2 RNA molecules provides a new paradigm for infection dynamics and variant differences. *Elife* **11** (2022).

6. E. R. Watkiss, P. Shrivastava, N. Arsic, S. Gomis, S. van Drunen Littel-van den Hurk, Innate and adaptive immune response to pneumonia virus of mice in a resistant and a susceptible mouse strain. *Viruses* **5**, 295–320 (2013).

Supplementary Table 1: Key Resources

**Cell lines**	**Source**
Calu-3	Kind gift from Professor Nicole Zitzmann
HEK293-p125	Professor Jan Rehwinkel
HEK293-ISRE	Professor Jan Rehwinkel
HEp-2	Kind gift from Dr Dalan Baley
Vero	Kind gift from Professor William James
RCC4	Kind gift from Professor David Mole
786-0	Kind gift from Professor David Mole
BHK-21	Kind gift from Professor James Stewart
Primary bronchial epithelial cells	Lifeline Technologies
**Viruses**	**Source**
GFP-RSV	Kind gift from Magdalena A. Krzyzaniak
Human RSV Subtype A2	Kind gift from Magdalena A. Krzyzaniak
PVM J3666	Kind gift of Prof Andrew Easton (University of Warwick)
**Antibodies**	**Source**
Mouse anti-HIF-1α	BD Transduction Laboratories, Clone 610959
Rabbit anti-NDRG1	Cell Signalling, Clone 5196
Mouse anti-β-actin	Sigma, clone#A5441
Mouse anti-RSV-F antibody	Made in-house, PMID:1383403
Rabbit anti-VHL	Cell Signaling #68547<br>Proteintech 24756-1-AP
Rabbit anti-ISG15	Abcam ab285367
Mouse anti PVM-G	Kind gift of Prof Andrew Easton (University of Warwick)
Rabbit anti-Iba1	WAKO Pure Chemical Industries, Ltd, 019-19741
Rabbit anti-CD3 (clone SP7)	Abcam ab16669
Rat anti-Ly6G (clone 1A8)	BioLegend 127602
Rabbit anti-mouse IgG	Abcam ab125904
Mouse anti-m^6A	Proteintech 68055-1-IG-20UL
**Chemicals and Commercial Assays**	**Source**
qPCRBIO SyGreen Blue Mix	PCR Biosystems Ltd PB20.17-20
Firefly Luciferase Assay kit	Promega E1501
Daprodustat (GSK:1278863)	MedChemExpress HY-17608
IFN alpha-IFNAR-IN-1	MedChemExpress HY-12836A
**Cells**	**Source**
Calu-3	Kind gift from Professor Nicole Zitzmann
HEK293-p125	Professor Jan Rehwinkel

HEK293-ISRE	Professor Jan Rehwinkel
HEp-2	Pirbright Cell Service Unit
Vero	Kind gift from Professor William James
RCC4	Kind gift from Professor David Mole
786-0	Kind gift from Professor David Mole
BHK-21	Kind gift from Professor James Stewart
Primary bronchial epithelial cells	Lifeline Technologies
**Oligonucleotides**	**Sequence (5'-3')**
B2M For	CTACACTGAATTCACCCCCACTG
B2M Rev	ACCTCCATGATGCTGCTTACATG
NDRG1 For	TTTGATGTCCAGGAGCAGGA
NDRG1 Rev	ATGCCGATGTCATGGTAGGT
RSV-N For	GCAGGATTGTTTATGAATGCC
RSV-N Rev	CCTCAACAACTTGTTCCATTTC
ISG15 For	CTCTGAGCATCCTGGTGAGGAA
ISG15 Rev	AAGGTCAGCCAGAACAGGTCGT
CA9 For	TATCTGCACTCCTGCCCTCTG
CA9 Rev	CACAGGGTGTCAGAGAGGGTGT
PVM-N For	ATGCTGCCAAAGATTATGCA
PVM-N Rev	ATCAACAATGTTCAGCTGCT
PVM-NS2 For	CGAGCCTACAAAACATCACT
PVM-NS2 Rev	TCTTCCACATATTGCTTGGC
Mouse ISG15 For	CATCCTGGTGAGGAACGAAAGG
Mouse ISG15 Rev	CTCAGCCAGAACTGGTCTTCGT
Mouse IFNB1 For	GCCTTTGCCATCCAAGAGATGC
Mouse IFNB1 Rev	ACACTGTCTGCTGGTGGAGTTC
Mouse Edn1 For	CTACTTCTGCCACCTGGACATC
Mouse Edn1 Rev	CGCACTGACATCTAACTGCCTG
**PVM smFISH Oligonucleotides**	**Sequence (5'-3')**
PVM-N-P-1	CCCACAAGGTACACGGAG
PVM-N-P-2	GGCCTCTCCTAGTATTAT
PVM-N-P-4	TGTGGGGTCAATGGGAGG
PVM-N-P-6	GTCCTACCCTTTGTTTTA
PVM-N-P-7	GGCAGGTTTAGAGATGGT
PVM-N-P-8	CTCTGTGTGGTACGGGAC
PVM-N-P-9	GCAAATCAAGCCACCTCT
PVM-N-P-10	GCTCGGCTGGAACTTTTC
PVM-N-P-14	ACTTTGGGTATTTCACCC
PVM-N-P-16	GGAATGCATCCTGTGCAC

PVM-N-P-17	ATCAAGTCATGGCCTGGG
PVM-N-P-19	CGGCCTAGGATGTGTATT
PVM-N-P-20	CAGTTGGACAGCAGGCTA
PVM-N-P-21	TACATCGCCTGTGGATCT
PVM-N-P-22	GGGCTTTCTGCATAGCAT
PVM-N-P-23	AGAACATGCCGAGTGTCC
PVM-N-P-25	GCATATTGGAGCCCTATT
PVM-N-P-26	ATGCTATCATCTCTGCCT
PVM-N-P-27	TTTACATTGTAGCCGGCT
PVM-N-P-28	CTGCATCTATTCCCACTA
PVM-N-P-32	CCCTGCAGCTAATTTGGA
PVM-N-P-33	TTCCACAGCATCAAGTCC
PVM-N-P-34	CTCGGCTTTCAGCACATT
PVM-N-P-35	TGCTTGACCTCCATGTTG
PVM-N-P-38	AGACCTGAAAACAGCCCC
PVM-N-P-40	ATTTTGCCAGTAAACCCC
PVM-N-P-41	CATGGCCTAGCATGATGT
PVM-N-P-42	CCATCTCAGCTTGTACAC
PVM-N-P-43	CGTAAACCTCAACCACCT
PVM-N-P-46	TCTATTCCTAGGAGCACC
PVM-N-P-48	CAATGTTCAGCTGCTGGC
